# Use of Ionic Liquids and Deep Eutectic Solvents in Polysaccharides Dissolution and Extraction Processes towards Sustainable Biomass Valorization

**DOI:** 10.3390/molecules25163652

**Published:** 2020-08-11

**Authors:** Eduarda S. Morais, André M. da Costa Lopes, Mara G. Freire, Carmen S. R. Freire, João A. P. Coutinho, Armando J. D. Silvestre

**Affiliations:** Department of Chemistry, CICECO—Aveiro Institute of Materials, University of Aveiro, 3810-193 Aveiro, Portugal; morais.eduarda@ua.pt (E.S.M.); andremcl@ua.pt (A.M.d.C.L.); maragfreire@ua.pt (M.G.F.); cfreire@ua.pt (C.S.R.F.); jcoutinho@ua.pt (J.A.P.C.)

**Keywords:** polysaccharides, solubilization, extraction, ionic liquids, deep eutectic solvents

## Abstract

A shift to a bioeconomy development model has been evolving, conducting the scientific community to investigate new ways of producing chemicals, materials and fuels from renewable resources, i.e., biomass. Specifically, technologies that provide high performance and maximal use of biomass feedstocks into commodities with reduced environmental impact have been highly pursued. A key example comprises the extraction and/or dissolution of polysaccharides, one of the most abundant fractions of biomass, which still need to be improved regarding these processes’ efficiency and selectivity parameters. In this context, the use of alternative solvents and the application of less energy-intensive processes in the extraction of polysaccharides might play an important role to reach higher efficiency and sustainability in biomass valorization. This review debates the latest achievements in sustainable processes for the extraction of polysaccharides from a myriad of biomass resources, including lignocellulosic materials and food residues. Particularly, the ability of ionic liquids (ILs) and deep eutectic solvents (DESs) to dissolve and extract the most abundant polysaccharides from natural sources, namely cellulose, chitin, starch, hemicelluloses and pectins, is scrutinized and the efficiencies between solvents are compared. The interaction mechanisms between solvent and polysaccharide are described, paving the way for the design of selective extraction processes. A detailed discussion of the work developed for each polysaccharide as well as the innovation degree and the development stage of dissolution and extraction technologies is presented. Their advantages and disadvantages are also identified, and possible synergies by integrating microwave- and ultrasound-assisted extraction (MAE and UAE) or a combination of both (UMAE) are briefly described. Overall, this review provides key information towards the design of more efficient, selective and sustainable extraction and dissolution processes of polysaccharides from biomass.

## 1. Introduction

The increased interest and need for sustainable growth of the world population as well as the concerns related to massive pollution and climate changes have led to switch the paradigm of a fossil-based economy to a sustainable bio-based model of development. In the present decade, a progressive shift from petroleum-derived products has been a target, but also a challenge placed by the European Union that has been recommending and encouraging the utilization of renewable resources for the production of a wide range of commodities [1]. It is estimated that the share of bio-based chemicals will continue to grow and that at least 30% of the chemicals will be derived from renewable resources by 2050 [2]. The production of chemicals from such feedstocks is expected to reach 113 million tons by the same year, corresponding to 38% of all organic chemicals production [3]. Therefore, the processing of renewable resources with increased valorization holds an enormous potential to cover future economic and environmental challenges.

The valorization of biomass feedstocks has been addressed in recent decades through the application of conventional technologies that often use hazardous solvents/reagents [4], and thus lack a more safe and sustainable exploitation of those raw materials. Therefore, there is an urgent need to replace these technologies by innovative processes based on green principles and capable to promote an efficient biomass fractionation and valorization. Furthermore, these processes should be integrated into biorefinery platforms to achieve higher sustainability and increased economic outcomes. 

Lignocellulosic biomass represents the most abundant source of renewable feedstocks with an approximate production of 1.1 × 10^11^ tons/year, consisting of non-edible plant materials, such as woody trees, shrubs and grasses [5,6], produced by forestry and agricultural sectors. The composition of this biomass varies with several factors but it is mainly composed of polysaccharides, such as cellulose, hemicelluloses, starch and lignin, as well as, in smaller amounts, of other components like proteins and extractives such as terpenes, sterols, phenolics and fatty acids, among many others [7].

Food waste is also abundant and an underexploited source of biomass. According to the Food and Agriculture Organization (FAO), one third of the food produced for human consumption is lost (all food that is not sold and/or not consumed) or wasted (processing residues), representing 1.3 billion tons/year of “food losses” and processing waste [8]. The enormous diversity of structures and macromolecules that can be found in food waste represent a potential source for added value compounds [9,10]. For instance, fruit peels, which have been merely used as animal feed, brought to landfills and sent for composting [11], represent a major resource of two polysaccharides, namely pectins and starch. On the other hand, marine food waste such as crustacean shells is rich in chitin, which is considered a polysaccharide with high added value (forecast to reach USD 2941 million by 2027) [12] and exploitation potential, being used in many biomedical and industrial applications [13]. In addition, the generation of food waste has raised social and environmental constraints, which can be overcome through the right implementation of well-designed food waste valorization chains. Since most of these residues present low economic value, their processing and fractionation could be an interesting market opportunity to bring new economic and ecological benefits [14].

**Figure 1:**
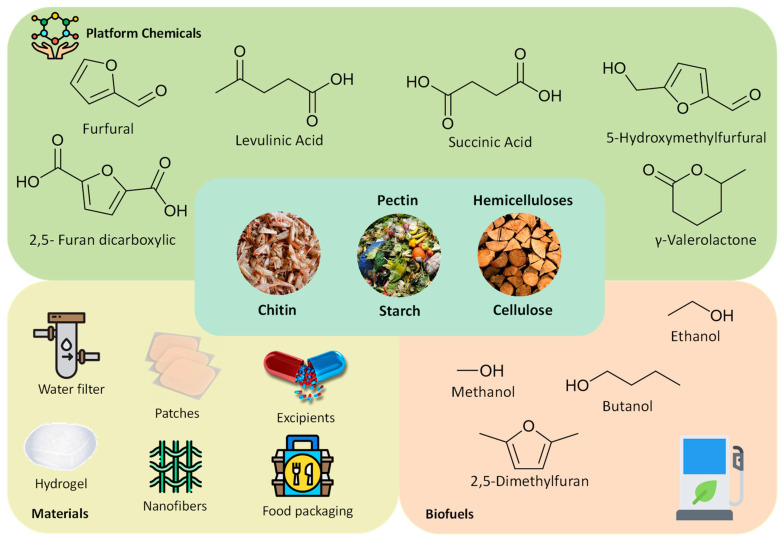
Examples of applications and products from biomass polysaccharides.

Polysaccharides have been prompting high interest as raw materials for different purposes (Figure 1). Polysaccharides can be depolymerized into monomers such as glucose, xylose and fructose, and converted into biofuels and building block chemicals. Bioethanol and biobutanol are two biofuels that can be produced from lignocellulosic polysaccharides and are good examples of large energetic commodities that society plans to embrace in a near future [15]. Furthermore, several chemicals, including 4-diacids (succinic, fumaric and malic), 2,5-furandicarboxylic acid, 3-hydroxypropionic acid, aspartic acid, glucaric acid, glutamic acid, itaconic acid, levulinic acid, 3-hydroxybutyrolactone, glycerol, sorbitol and xylitol/arabinitol can be obtained from polysaccharides. These compounds are extremely important and have been identified as the top 12 carbohydrate-derived chemicals by the US Department of Energy [16]. On the other hand, i.e., instead of performing their disassembly and cleavage into monomers, polysaccharides as natural polymers have intrinsic features to be applied in the design and synthesis of novel polymeric materials [17]. Polysaccharides have been extensively applied in the development of different functional materials, as stimuli-responsive materials (gels) [18], films for water filtration [19,20], functional textiles [21] and electrochemical materials (membranes) [6], among many others.

Polysaccharides have a wide variety of chemical structures and, therefore, a varied assortment of properties. In this sense, it is imperative to deeply understand their structure and properties to successfully use them or to select those more suited to the desired application [22].

### 1.1. Polysaccharides in Lignocellulosic Materials and Food Wastes

The main polysaccharides found in biomass resources are cellulose, chitin, starch, hemicelluloses and pectins, whose chemical structures are displayed in Table 1. Cellulose and hemicelluloses are the main polysaccharide fractions of lignocellulosic biomass and can reach as high as 80 wt% of the total biomass content. Small amounts of pectins and starch are also present in lignocellulosic materials [7], but these are much more abundant in fruit and vegetable waste [11]. Finally, chitin is a structural component found in crustacean shells, which are one of the predominant marine disposals and wastes from the shellfish industry at the present [23]. The main structural features of these polysaccharides are also depicted in Table 1.

Cellulose is a highly crystalline homopolysaccharide composed of repeated glucose units linked by β(1→4) glycosidic bonds and corkscrewed 180° with the respective neighbor unit, with the dimer repeated segment being called cellobiose [24]. The β configuration at the anomeric carbon of each glucose unit gives rise to a stretched chain conformation, while hydrogen bond interactions between hydroxyl groups of anhydro glucose units allow these chains to be arranged into flat sheets [25,26]. The high crystallinity is due to the formation of an intramolecular hydrogen bond network extending from the C_3_ hydroxyl to the C_6_ hydroxyl group of the next unit across the glycosidic linkage and from the C_2_ hydroxyl to the C_6_ hydroxyl of the next residue [24]. This hydrogen bonding network creates long fibers organized in a hierarchy of elementary fibrils (1.5 and 3.5 nm), microfibrils (10 and 30 nm) and microfibrillar bands (100 nm) [27]. The high crystallinity and fiber morphology are structural features that make cellulose an insoluble material either in water or in most conventional organic solvents. Cellulose has been used mostly for pulp and paper production and in the textile industry, but its inherent properties turn it into an outstanding bio-based material for a myriad of applications, such as an additive in the food industry, production of conductive nanofibers in nanotechnology, films with bioactive compounds in cosmetics, drug delivery systems for pharmaceuticals, biodegradable films for food packaging, clothing and microspheres for catalyst immobilization [28,29,30,31,32,33,34,35,36,37].

Hemicelluloses are branched amorphous heteropolysaccharides [3], representing 20–35 wt% of lignocellulosic biomass. The structure and composition of hemicelluloses, including the type of composing monosaccharides, type and position of chemical linkages, the presence of side groups/chains and their distribution, strongly vary with the biomass source [38]. Some key examples of hemicelluloses are xylans, mannans, xyloglucans and mixed linkage β-glucans [39]. In hardwoods, for example, glucuronoxylans (represented in Table 1 as *O*-acetyl-4-*O*-methyl-glucurono-β-d-xylan) are the predominant hemicellulose structures [40]. In contrast, glucomannan, arabinoglucuronoxylan and arabinogalactan are mostly found in softwoods [41]. The structural and compositional variability associated to hemicelluloses give them interesting physicochemical features in the development of novel bio-based materials, like biomedical scaffolds [42], coatings [43], plasticizers [44], films and binders [45], flocculants or hydrogels [46]. Hemicelluloses can also be used as feedstock for conversion into biofuels and different building block chemicals [47,48].

Starch is a semi-crystalline polysaccharide [49] composed of amylose and amylopectin, which are arranged in water-insoluble granules of different size (1 to more than 100 µm). The size, morphology and composition of these granules vary with the biomass source as well as with the isolation method [50]. Both amylose and amylopectin are composed of glucose units linked by glycosidic α(1→4) bonds, with amylopectin being slightly branched and thus presenting α(1→6) linkages. Starch has a helical shape due to the α configuration of the anomeric carbon [25]. Due to its granular and semi-crystalline structure, starch has low solubility in most common solvents. Nonetheless, when heated in water, starch undergoes an order–disorder endothermic transition called gelatinization. This phenomenon enables starch granules to absorb water and swell, while amylose is leached out of the granules, disrupting the semi-crystalline structure [51]. On the other hand, when exothermic transition occurs, starch is dissolved without gelatinization, meaning that the hydrogen bonds in the starch structure are broken and the granules are completely disrupted and dissolved in the solvent [52,53]. Since glucose is the monomeric unit composing starch, similar applications can be adopted for starch conversion as those applied for cellulose, including the production of the same building block chemicals [47]. Furthermore, bio-based materials for food, paper, cosmetic, adhesive and pharmaceutical applications can be produced using starch [21,54,55,56,57]. However, for most applications, starch lacks the required properties due to its semi-crystalline structure. Therefore, starch is generally transformed into thermoplastic starch (TPS) mostly by using water as a plasticizer coupled with heating and shear treatments, causing its destructuration [58]. Nevertheless, starch can suffer retrogradation, i.e., it can re-organize in its semi-crystalline structure even after the gelatinization process. This becomes more pronounced when starch is dispersed in water followed by solvent evaporation. To avoid these issues, glycerol or other polyols are added as plasticizers, increasing the flexibility and processability of the polymer. Nonetheless, some TPS mechanical properties, namely elongation and tensile strength, can be affected due to leaching of these plasticizers over time as consequence of their small molecular size [58,59].

Pectins are a semi-crystalline polysaccharide commonly present in by-products of the fruit juice industry [60] and are a complex family of polysaccharides mostly composed of 1,4-linked α-d-galacturonic acid (GalpA) units. Furthermore, these polysaccharides are generally classified according to their degree of esterification (DE), i.e., the number of carboxylic acid groups from the galacturonic acid residues esterified with methyl groups. The DE will influence characteristics such as the gelation mechanism and also the processing conditions of these polysaccharides. Pectins with high DE require the presence of either sugar or acid for gelation to occur, due to the occurrence of hydrogen bonding and hydrophobic interactions between the pectin chains. On the other hand, low-ester pectin can gel in the presence of cations such as Ca^+^ and in this case the affinity of pectin chains increases with a decrease in DE [61,62]. Pectins can be divided in four groups according to their structure and ramifications: (i) homogalacturonan (HG), also known as the “smooth region”; (ii) xylogalacturonan (XGA); (iii) rhamnogalacturonan I (RGI), known as the “hairy region”; and (iv) rhamnogalacturonan II (RGII) [63,64,65]. The extraction of pectins from food waste along with further valorization into new products has been addressed, especially in the food industry field [65]. Pectins can be used in many applications, such as gelling and stabilizing emulsions for cosmetic purposes or nutraceutical formulations [66], as well as in cancer prevention and treatment [67]. In addition, pectins can be applied as raw materials for the production of special and edible films and adhesives [68], paper additives, plasticizers [60], biomedical devices and materials for drug delivery [62].

Chitin is a semi-crystalline polysaccharide that occurs as ordered crystalline microfibrils oriented in three different forms, namely antiparallel (α), parallel (β) and alternating (γ) orientations [69,70]. This polysaccharide is composed of 2-acetamido-2-deoxy-d-glucose units and its deacylated form 2-amino-2-deoxy-d-glucose. The percentage of the 2-acetamido-2-deoxy-d-glucose units in chitin chains represents the degree of acetylation (DA). Chitin typically presents a DA near 0.90, which has direct impact on the polysaccharide properties and solubility [71]. Chitin, is highly insoluble in water and rigid, acting as a structural polysaccharide in living organisms [13]. The solubility limitation is associated to the intrinsic high DA and to its fibrillar organization. Therefore, deacetylation is generally utilized producing its *N*-deacetylated form named chitosan. Although the *N*-deacetylation is never complete [13], chitosan generally has DA values lower than 0.50 [72].

Chitin is mainly used as a chelating agent for metal ions, due to the electron pair available on nitrogen atoms in acetamido groups and on oxygen atoms in hydroxyl groups. Immobilization of enzymes and cells, wound dressing and suturing agent, excipient and binder formulations are additional examples of chitosan applications [73]. Like other polysaccharides, chitin can be depolymerized and converted into pivot chemicals, especially aminated compounds, such as 3-acetamido-5-acetylfuran (3A5AF) [74], amines and amine polyols [75]. Chitosan, on the other hand, is mainly used for the production of innovative materials comprising capsules, films and hydrogels for applications as drug delivery systems and scaffolds for tissue engineering [76,77]. Chitosan-based materials are also widely used in catalysis and water remediation [78,79].

All the chemical structures, chemical composition, degree of polymerization and type of linkages for each of the mentioned polysaccharides are compiled in Table 1. Moreover, the type of biomass in which each polysaccharide is abundant is also described.

### 1.2. Alternative Solvents as Tools for Polysaccharides Dissolution and Extraction

Considering the potential of polysaccharides highlighted above, their extraction from biomass is of upmost importance. Conventional extraction processes usually contemplate the use of unsustainable and volatile organic solvents, their mixtures or other harsh acidic or alkaline aqueous media, capable of swelling and hydrating biomass and to facilitate mass transfer of the soluble constituents from the solid matrix to the solvent [83]. The combination of dimethyl acetamide (DMAc) with lithium chloride to dissolve cellulose [84,85] or dimethyl sulfoxide (DMSO) used for starch dissolution [86] are some examples. Chitin, meanwhile, can be solubilized in acids such as dichloroacetic (DCA) and trichloroacetic (TCA) but also in DMAc with lithium chloride [6,87]. On the other hand, harsh acidic or alkaline conditions are commonly used in the dissolution and extraction of hemicelluloses from lignocellulosic biomass [88], demonstrating a lack of sustainability. 

Coupled with those disadvantages, conventional extractions not only are highly energy-intensive, requiring high temperatures [89,90,91,92] or overall biomass pre-treatment [7], but also exhibit low selectivity [25,93]. As described above, the development of novel, efficient and sustainable extraction technologies is a key of success, but also a challenge that must be addressed. The use of alternative solvents and technologies capable to compete with the conventional organic solvents has become a critical challenge for the scientific community [1,94]. Two types of solvents have gained increased interest in this realm, namely ionic liquids (ILs) and deep eutectic solvents (DESs). Furthermore, coupled with specific technologies, such as microwave and ultrasound (MAE and UAE, respectively), the performance of these solvents can be improved.

**Figure 2:**
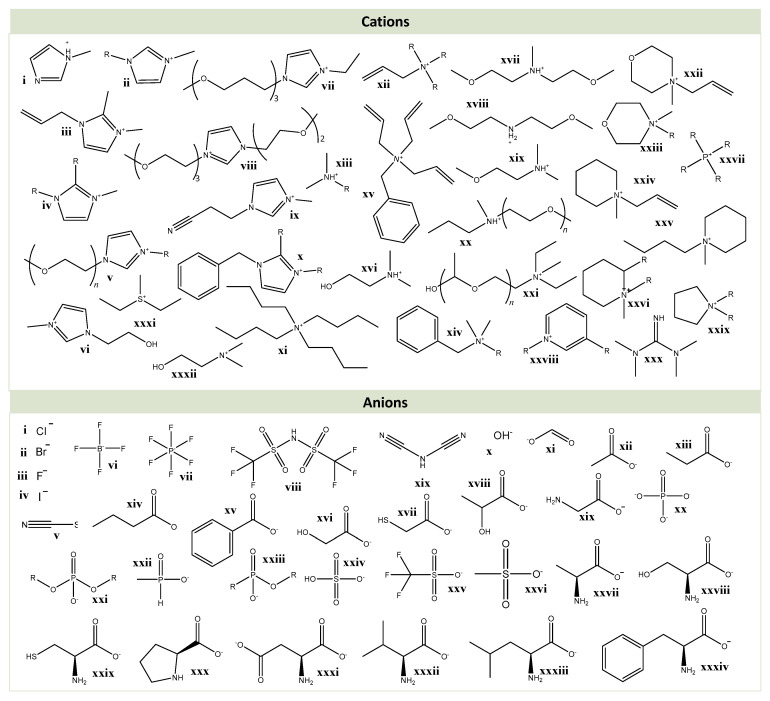
Chemical structures of the IL cations and anions reviewed in this work.

Ionic liquids (ILs) (Figure 2) are salts generally composed of a large organic cation and a smaller organic or inorganic anion, possessing a melting temperature below 100 °C. Their low melting temperature is governed by their low charge density and low symmetry ions in their composition [95]. ILs are also described as “designer solvents” due to the numerous combinations of the existing cations and anions that can form an IL, allowing to tailor them for specific applications [96]. In general, if properly designed, ILs present several interesting properties, such as electrical conductivity, negligible vapor pressure, low flammability, tunability and excellent thermal stability, and are good solvents for a wide range of polar and non-polar compounds. The negligible vapor pressure and high thermal stability stand out as excellent advantages over conventional volatile organic solvents [95]. Furthermore, ILs may be less toxic than traditional volatile organic solvents, although a comprehensive toxicity evaluation is still required [97]. Nevertheless, due to their “designer solvents” feature, it is possible to design ILs with low toxicity [98]. Actually, ILs containing more biocompatible cations and anions, derived from relatively inexpensive and eco-friendly natural resources, such as carbohydrates and amino acids, have been emerging. Cholinium-based ILs, whose cation can be found in nature, are a major example of ILs with low toxicity [1]. ILs began to be applied as electrolytes in battery applications in 1970 [99] and their applications rapidly shifted to several fields, such as organic chemistry and biochemistry [100], biomass fractionation [25,101], extraction of carbohydrates [5,94,102,103] and other biomolecules [96]. Additionally, ILs have been used as biomass delignification media as they are able to remove lignin and to expose the carbohydrate fraction for enzymatic hydrolysis [104]. In addition, some ILs have been reported to completely dissolve biomass [101].

Figure 2 and Table 2 present all the IL cations’ and anions’ descriptions, nomenclature and chemical structures that will be mentioned in this review, used either in the dissolution or extraction of polysaccharides.

**Figure 3:**
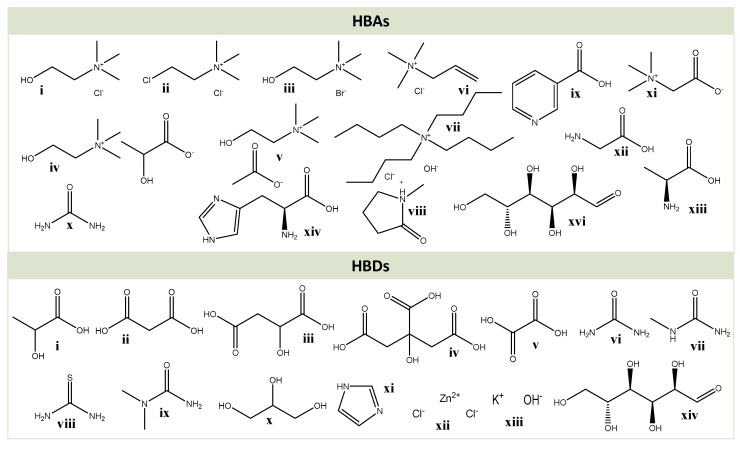
Chemical structures of the DESs’ HBDs and HBAs reviewed in this work.

Deep eutectic solvents (DESs) were first reported by Abbott et al. [105] as mixtures of solid compounds that form a eutectic mixture with a lower melting point than each of the individual components. This melting point depression can be attributed to the hydrogen bonding network established amongst components and to the charge delocalization resulting from it [106,107]. In this context, DESs are composed of at least one hydrogen bond donor (HBD) and one hydrogen bond acceptor (HBA) (Figure 3) and can be easily prepared by mixing both components in certain molar ratios. Moreover, if properly designed, they are cheap to produce and possess low toxicity, especially those derived from renewable resources [108].

The applications of DESs have evolved during the years, from enzymatic catalysis to drug delivery, and more recently, their application in biomass fractionation and extraction has been approached [106]. Several studies have reported the potential of DESs towards lignin dissolution [109] and depolymerization [110,111,112], as well as polysaccharides dissolution (e.g., starch and chitin) [113,114]. DESs based on carboxylic acids (e.g., oxalic, lactic and glycolic acids) and polyols (e.g., ethylene glycol, 1,2-propanediol and glycerol) have shown the ability to remove lignin and to separate xylans from biomass [115,116,117,118]. The names, acronyms and structures of the HBAs and HBDs of the different DESs mentioned in this review can be found in Table 3 and Figure 3.

In this review, a comparative discussion about the application of new and innovative solvents (ILs and DESs) for polysaccharide dissolution and extraction is presented. In this work, only the dissolution and extraction of cellulose, hemicelluloses, starch, pectins, chitin and chitosan assisted by ILs and DESs as neoteric solvents are considered.

**Figure 4:**
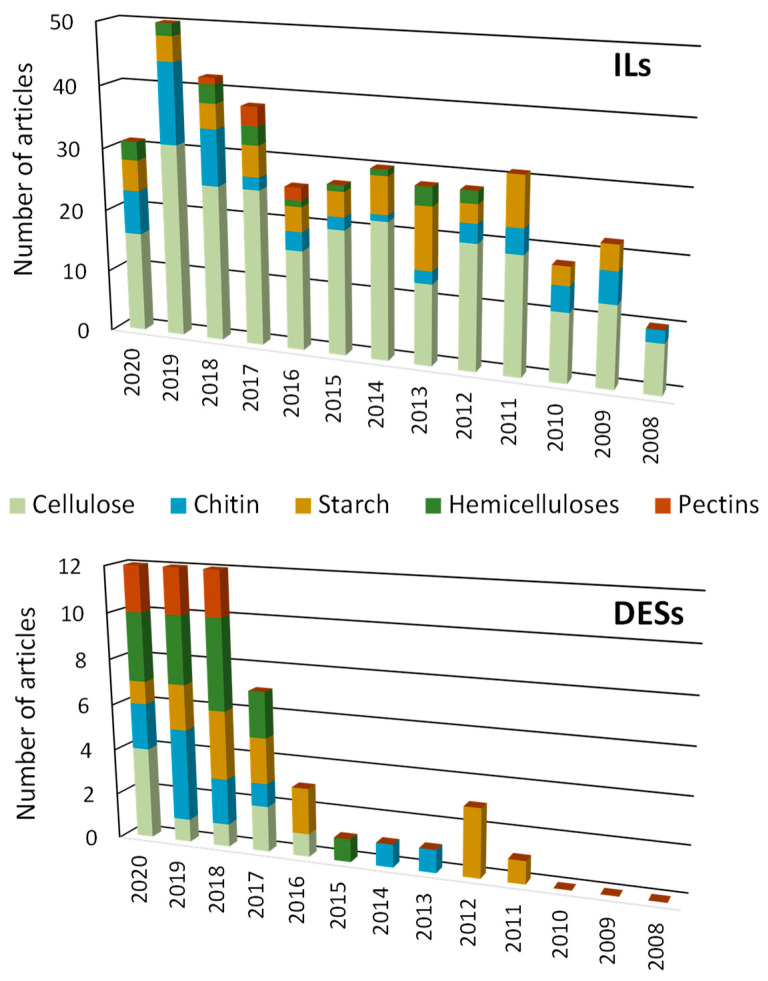
Number of articles published per year regarding the dissolution and extraction of the different types of polysaccharides covered in this review using ILs and DESs.

Figure 4 summarizes the growth of the scientific papers related to this subject divided by type of solvent and polysaccharide. As expected in the field of ILs, cellulose has the higher bulk of scientific papers published, followed by chitin and starch. The number of papers published follows closely the abundance of each polysaccharide in nature, being hemicelluloses and pectins less studied due to their lower abundance in nature. Nonetheless, hemicelluloses have started to gain more interest in recent years. DESs, on the other hand, do not present the same tendency as ILs. Firstly, the bulk of work is much lower when compared with ILs, which is in accordance with their novelty. Moreover, the first works published using DESs focused mostly on starch and chitin instead of cellulose, which only began to be studied in 2016. The studies with DESs also focus more on pectins and hemicellulose, which could be a consequence of the poor results achieved with ILs with these two polysaccharides.

In addition to the works overview in this field, the fundamental chemistry behind the dissolution and extraction of polysaccharides in these green solvents is scrutinized. Nonetheless, in the cases where the solvents do not allow a selective solubilization and extraction of the polysaccharide, the process is analyzed and comparatively discussed. Furthermore, synergies on the use of ILs and DESs with non-conventional technologies, such as MAE and UAE, are here presented and discussed.

## 2. Cellulose

**Figure 5:**
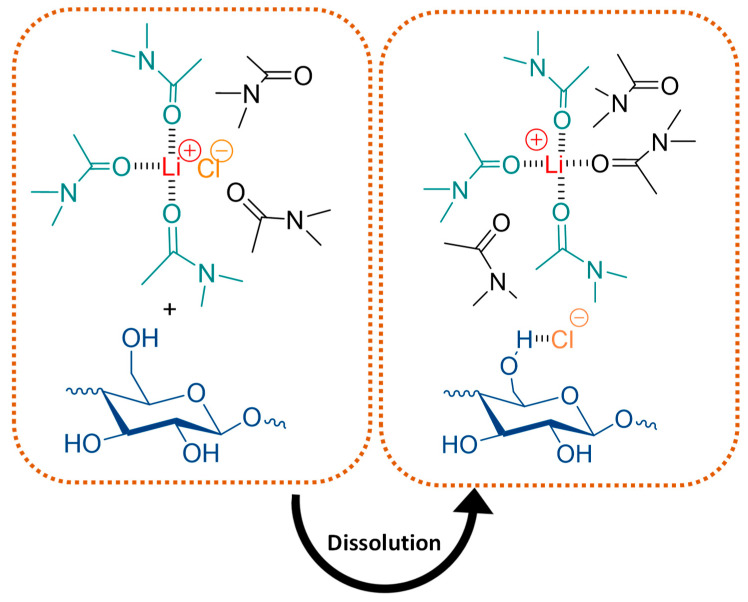
Schematic representation of the interactions among Li^+^ cation, Cl^−^ anion and DMAc when cellulose dissolves into the DMAc/LiCl system. Adapted from [122].

The conventional process for the dissolution of cellulose usually requires the activation of the cellulose structure by opening the polymer chain in a more relaxed conformation [119]. The standard method for cellulose dissolution explores the use of dimethylacetamide (DMAc). The dissolution mechanism involves the formation of hydrogen bonds between cellulose and a DMAc/lithium chloride (LiCl) complex [84,85]. Two different approaches can be performed according to the pre-treatment step applied: (i) thermal treatment or (ii) polar medium swelling. In the first case, cellulose is pre-treated with hot DMAc at 150 °C, followed by the addition of LiCl with a maximum concentration of 10 wt% (the solubility limit of this salt in DMAc) [84,90,91]. The solution can become slightly colored due to the partial oxidation of cellulose at high temperature [85]. On the latter (ii), cellulose is pre-treated with water to swell and open the crystalline structure. Solvent exchange is then done with DMAc/LiCl solution and, in some cases, methanol is also employed as an extra step before the use of DMAc [119,120]. The dissolution mechanism has been explained by McCormick et al. [121]: ion–dipole complexes are formed between Li^+^ and the carbonyl group of DMAc and hydrogen bonds occur between Cl^−^ and hydroxyl groups of cellulose (Figure 5) [85,122]. This was further confirmed in other studies that referred cellulose hydroxyl protons to established strong hydrogen bonds with the Cl^−^ anion. This hydrogen bonding network results from the disruption of the intermolecular hydrogen bonding of cellulose and simultaneous splitting of the Li^+^–Cl^−^ ion pairs [122].

Cellulose solutions in DMAc are highly stable, showing no degradation for several months, especially with higher LiCl concentrations [119,120]. Overall, the polar medium swelling method is the most efficient, achieving up to 15 wt% cellulose solubility within 48 h or less, but this capacity refers to low-molecular weight cellulose fibers. However, some authors recognize the heating method as better, since less LiCl is required and no solvent exchange is needed [123]. The careful preparation of this solvent system must be taken in account since both DMAc and LiCl are hygroscopic and water will hinder the complexation with cellulose. In solvent preparation, the water content should be lower than 5 wt% [84].

Industrially, *N-*methylmorpholine-*N*-oxide (NMMO) is preferably chosen to promote cellulose dissolution. This solvent was first introduced in early 1980 and is now employed in the lyocell process [124]. NMMO has a high capacity to form hydrogen bonds, is a strong oxidant and is slightly basic. The NMMO solvation power arises from its ability to form complexes with water and cellulose macromolecules. The process occurs at temperatures between 72 and 120 °C with 4–17 wt% water content. Under these conditions, NMMO·H_2_O can breach the cellulose hydrogen bonds and establishes a NMMO dipole complex with cellulose hydroxyl groups, allowing up to 30 wt% of cellulose dissolution, depending on the pulp characteristics [89,121].

More recently, the dissolution of cellulose in a mixture of urea/sodium hydroxide (NaOH)/water has been introduced by Zhang and co-workers [125]. These results contradict the notion that water is detrimental to cellulose dissolution as shown in the DMAc process. The dissolution of 5 wt% cellulose is achieved at 12 °C with aqueous solutions containing 7 wt% NaOH and 12 wt% urea, and takes place in a few minutes [125]. The dissolution mechanism involves the formation of a hydrogen bonding network between the hydroxyl groups and clusters of NaOH/water at low temperature. Afterwards, urea forms a shell around the clusters, the so-called inclusion complex (IC), leading to the dissolution of cellulose [126]. This mechanism is also visible in a similar solvent mixture, an aqueous solution of lithium hydroxide (LiOH)/urea. Approximately 4 wt% cellulose dissolution was reported for both LiOH/urea and NaOH/urea at −10 °C [126].

Other methodologies have also been adopted, but they usually lead to the degradation of cellulose. For example, concentrated aqueous solutions of phosphoric acid (>80%) have also been used for cellulose dissolution [127], although it is well known that polysaccharides, and obviously cellulose, are prone to hydrolysis under acidic conditions, therefore this process might also have a substantial impact on the dissolving pulp (DP) of the polysaccharide. Another example comprises the use of sodium hydroxide aqueous solutions (NaOH). Although it has led to good results in dissolving low-molecular weight cellulose [121] and amorphous cellulose (4 wt% solubility) [128], and although polysaccharides are substantially more stable in alkaline than in acidic conditions, the so-called peeling reactions [129] can still take place and result in DP decrease. However, this effect can be reduced as shown by Navard and co-workers [130], who demonstrated that a combination of NaOH and water with ZnO caused the formation of hydrates due to ZnO’s role as a water binder, which were then able to penetrate into the cellulose structure, but also stabilize cellulose against undesired gelation [130].

### 2.1. Cellulose Dissolution with Ionic Liquids

As previously highlighted, alternative solvents and technologies towards more sustainable ways of dissolving cellulose have been investigated. ILs are a promising class of solvents for this task. ILs such as 1-ethyl-3-methylimidazolium chloride ([C_2_C_1_im]Cl) and 1-ethyl-3-methylimidazolium acetate ([C_2_C_1_im][CH_3_COO]) have been reported as remarkable cellulose solvents, and the process can be improved in combination with mechanical treatments such as ball milling [101]. The particular relevance of [C_2_C_1_im][CH_3_COO] should be remarked since it is the only IL that can be commercialized in the European Union in volumes exceeding one ton per annum (tpa), since it is approved by Registration, Evaluation, Authorization and Restriction of Chemicals (REACH) [131].

Previous reviews have already highlighted the ideal characteristics of ILs to dissolve lignocellulosic biomass and cellulose [25,101,132]. Overall, ILs should be used in dry form since water hinders the dissolution process [5,25]; smaller cations and anions are preferred; and the hydrogen bond basicity of the anion should be high to establish strong intermolecular bonds with cellulose [25]. Accordingly, the ability of ILs for cellulose dissolution has been correlated with Kamlet–Taft parameters (α, β and π) of IL anions, particularly with the parameter β that corresponds to the hydrogen bond basicity of the IL anion. Therefore, for higher β values of the IL anion, the ability of IL to dissolve cellulose is also higher [5,25,101,132]. This ability is connected to the hydrogen bonding that can be established between the IL anion and the cellulose hydroxyl groups, leading to improved solubility (cf. discussion below).

The potentialities of ILs for cellulose dissolution have been addressed extensively in the literature [133,134,135,136,137], with the main results summarized in Table 4. However, it should be mentioned that the comparison between gathered data is often tricky, since different types of cellulose (e.g., microcrystalline cellulose (MCC), Avicel or pre-hydrolysis sulfate pulp) were used in those studies. Cellulose dissolution in ILs was first demonstrated by Rogers and co-workers [133]. Approximately 25 wt% cellulose from dissolving pulp (DP ≈ 1000) was dissolved in 1-*n*-butyl-3-methylimidazolium chloride ([C_4_C_1_im]Cl) assisted by microwave heating (MAE) [133].

Following this pioneering work, the mechanisms behind the dissolution process were investigated [102,133,135,136]. The mechanism of cellulose dissolution using ILs is dependent on the disruption of the hydrogen bonds between cellulose chains and the establishment of new hydrogen bonds between cellulose –OH groups and the IL anions [136]. Hence, the anion should have a high electron density, short alkyl chain and, if possible, no electron withdrawing group. The interactions between cellulose and anions were found to decrease as follows: Cl^−^ > [CH_3_COO]^−^ > [[(C_1_O)HPO_2_]^−^ > [SCN]^−^ > [PF_6_]^−^ in the presence of the [C_2_C_1_im] cation [136]. The IL anion capability of dissolving cellulose directly correlates with the Kamlet–Taft parameter β, which describes the IL anion hydrogen bond accepting strength. Although ILs usually have a high β value, it should be above 0.8 to promote cellulose dissolution [25]. Other works pointed out that the acetate anion is more effective than the chloride one [135,138]. For example, Payal et al. [138] evaluated and compared the cellulose dissolution in several [C_4_C_1_im]-based ILs by computational methods and experimental data. It was found that cellulose solubility in ILs varied in the following order of the anions: [CH_3_COO]^−^ > Cl^−^ > [BF_4_]^−^∼[PF_6_]^−^ [138]. The conflicting reports about the best anion for cellulose dissolution are perhaps related to the interactions between those anions and the IL cation (cohesive energy) that could influence cellulose dissolution. Furthermore, hydrophobic interactions between cellulose and IL cations might also occur [139] and must be considered. Crystalline cellulose has an amphiphilic structure, meaning that its non-polar structures can organize into hydrophobic sheets paired against another. Thus, it is crucial that the solvent used for cellulose dissolution may also have an amphiphilic behavior. This means that the IL cation could also have an important role in cellulose dissolution [139].

In the literature, there is a consensus that imidazolium is a good cation for cellulose dissolution [102,133,136,138]. For instance, studies regarding the interactions established between [C_2_C_1_im][CH_3_COO] and 1,4-linked β-d-glucose oligomers revealed that the imidazolium cation promotes van der Waals interactions with the glucose pyranose rings [140]. Lu et al. [141] evaluated the effect of the cation on cellulose dissolution by testing 13 ILs with different cations and the same anion. The results showed that acidic protons of cations can establish hydrogen bonds with the hydroxyl groups of cellulose and oxygen atom in the glycosidic bond, increasing cellulose solubility. On the contrary, these cations can also have a negative effect if they strongly interact with anions, if they possess large electronegative atoms (like nitrogen and oxygen) in the cationic backbone or if they have a large-sized group in the alkyl chain, such as a hydroxyl group. In the last scenario, the cation’s hydroxyl group is expected to compete with the IL anion for establishing hydrogen bonds with cellulose [141]. Nevertheless, this rule does not always apply, as shown by Zhang et al. [142]. By employing a molecular dynamic approach, the researchers found that the presence of oxygen in the cation may have a positive effect, if an electron withdrawing group is present in the alkyl chain, such as an allyl group (e.g., [aC_1_im]Cl). The latter will enhance the interaction with cellulose, increasing the electronegativity of the cation [142]. Therefore, ILs should present anions with strong hydrogen bond basicity, and cations with acidic protons and without electronegative atoms or large groups in the cation alkyl chain [141]. Moreover, cellulose solubility decreases for longer alkyl chains in both cations and anions [136]. In this sense, ILs, such as 1-allyl-3-methylimidazolium chloride ([aC_1_im]Cl), [C_2_C_1_im][CH_3_COO], [C_4_C_1_im][CH_3_COO] and 3-methyl-*N*-butylpyridinium chloride ([MNBuPy]Cl), have demonstrated high performance for cellulose dissolution [5,102,136]. Although not so promising as those comprising cations with acidic protons, ILs with the pyridinium and quaternary ammonium cations are also good candidates for cellulose dissolution if paired with IL anions with high hydrogen bond basicity, which further reinforces the more relevant role of the IL anion to dissolve cellulose [135]. These results are in agreement with all the required characteristics for an IL to dissolve cellulose as described above.

Additionally, a new class of distillable acid–base conjugated ILs was successfully attempted to dissolve cellulose [143,144,145]. Combinations of superbases, including 1,1,3,3-tetra-methylguanidine (TMG), 1,5-diazabicyclo-[4.3.0]non-5-ene (DBN) and 1,8-diazabicyclo[5.4.0]undec-7-ene (DBU) with different organic acids [146,147,148,149,150,151], were used to synthesize protic ionic liquids (PILs), which were tested for dissolution of eucalyptus cellulose pulp fibers. For instance, TMG-based ILs achieved 13 wt% cellulose solubility [151]. These PILs not only showed a strong ability for cellulose dissolution but also demonstrated to be easily recovered by distillation. Recovery and purity yields up to 99% were achieved through a distillation process at 45 °C [143]. These ILs also disclosed high capacity for CO_2_ capturing, which is a remarkable property to tune polysaccharide dissolution, modification and precipitation [144].

Other works using PILs for cellulose dissolution have been published [4]. Guazzelli and co-workers [152,153] have produced new protic and aprotic ILs based in levulinic acid as an alternative to acetate. These ILs presented comparable cellulose dissolution ability to acetate-based ILs. For instance, 1-ethyl-3-methylimidazolium levulinate ([C_2_C_1_im][Lev]) achieved 29 wt% cellulose solubility ([153], while 1,5-diazabicyclo[4.3.0]non-5-ene levulinate ([DBNH][Lev]) reached 20 wt% [152]. Other PILs composed of DBU and carboxylic acids have been also tested for cellulose dissolution with good results (see Table 4) [154]. The researchers observed that cellulose dissolution ability increased with the ILs’ density and with the increment in the difference between the pKa values of DBU and carboxylic acid. Moreover, the alkyl chain length of the carboxylate anion also plays an important role in cellulose dissolution, since longer chains prevent the interactions between the IL anion and cellulose hydroxyl groups [154]. On the other hand, ILs composed of ammonium-derived cations (ethanolamine, diethanolamine, triethanolamine, propan-1-olamine and diallylamine) and organic acid-derived anions (formic acid, acetic acid, malonic acid and citric acid) were unable to dissolve cellulose [142]. This was expected since these ILs do not present the general accepted requirements for cellulose dissolution mentioned above [4]. 

Although ILs have demonstrated excellent capacity for cellulose dissolution, their use in the neat state often brings some drawbacks, mainly related to their high viscosity that hinders the mass transfer phenomena and requires the use of high temperatures [173]. Therefore, recent works in which ILs were used as co-solvents with dimethyl sulfoxide (DMSO), γ-valerolactone (GVL) or *N,N*-dimethylmethanamide (DMF) to dissolve cellulose have been reported [174,175,176,177,178,179]. The data compilation of the different works of cellulose dissolution with ILs and co-solvents is given in Table 5.

Kostag et al. [174,180] investigated the effect of mixtures of quaternary ammonium ILs and DMSO and discovered that several mixtures could readily dissolve MCC, between 4 and 10 wt%. [180]. Furthermore, the researchers demonstrated that ILs possessing cations with benzyl groups (allylbenzyldimethylammonium acetate ([NAl_2_Bz_1_][COO]) and triallylbenzylammonium acetate ([NAl_3_Bz][COO]) could achieve higher cellulose dissolution than imidazolium counterparts, and that the dissolution process was dependent on cation volume, rigidity and hydrophobic interactions between the cation chains [180]. In a different work, they further elaborate that cation density can hinder cellulose solubilization and the conformational flexibility related to its alkyl side chains can promote cellulose solubilization since it facilitates the cation diffusion and interaction with cellulose chains [174].

The addition of GVL [175] and imidazole [176] as co-solvents of ILs increased the solubility of cellulose when compared with the pure ILs, namely [C_2_C_1_im][COO] and 1-butyl-3-methylimidazolium phosphonate. In the first case, 15 wt% of α-cellulose dissolution was achieved at 80 °C, while only 5 wt% of cotton fibers were dissolved at 90°C. The addition of GVL to [C_2_C_1_im][COO] allowed an increase in solubility (15 wt% at 80 °C) when compared with the pure IL. Moreover, the addition of GVL decreased the dissolution activation energy, thus having a higher dissolution rate, whereas mixing imidazole with [C_4_C_1_im][(C_1_O)(H)PO_2_] decreased the viscosity of the system without affecting the H bond acceptor capacity of the IL [175,176]. In another study, Sánchez et al. [179] tested microwave irradiation for cellulose dissolution in 1:1 (*w/w*) mixtures of [C_2_C_1_im][COO] and DMSO, achieving an increase between 21% and 57% of cellulose dissolution rate compared to the pure IL [179]. These results show that using biodegradable and non-toxic co-solvents such as GVL with ILs is an attractive approach because it not only increases the performance for cellulose dissolution, but also decreases the overall cost of the process, making it more sustainable. It should be, however, remarked that this is not a universal trend and co-solvents should be carefully addressed and evaluated. [175].

Although a wide number of studies resorting to cellulose dissolution using ILs have been reported, it should be mentioned that no study regarding the selective extraction and dissolution of cellulose from biomass has been reported yet. ILs have been showing selectivity for lignin removal from biomass rather than polysaccharide fractions. However, the fractionation of biomass with ILs has been achieved through the whole dissolution of raw biomass materials, followed by selective precipitation of main fractions through the addition of selective anti-solvents to the IL/biomass mixture [5,181,182]. A typical example was shown by Jiang and co-workers [183], who applied the IL [aC_1_im]Cl to completely dissolve wood chips, and then a mixture of DMSO/water was used to selectively precipitate cellulose while maintaining lignin and hemicelluloses in solution. This topic is not discussed in detail in the present review, but it is comprehensively and critically debated elsewhere [101,184].

### 2.2. Cellulose Regeneration in Ionic Liquids

**Figure 6:**
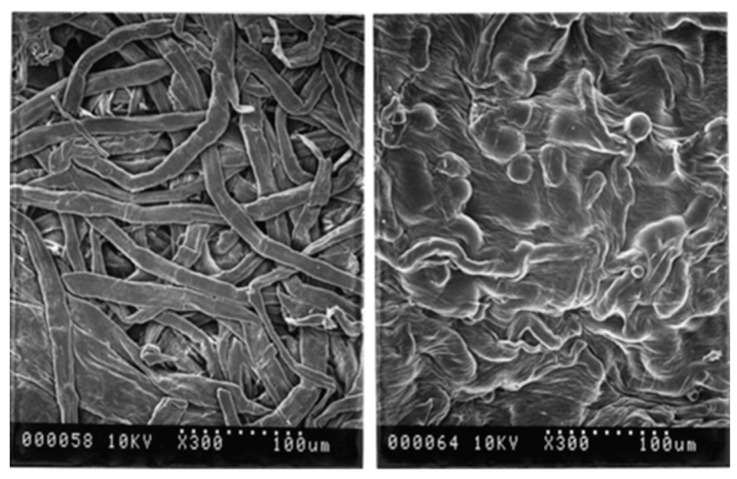
SEM micrographs of cellulose pulp fibers before (**left**) and after dissolution in [C_4_C_1_im] Cl (**right**). Reprinted with permission from [133]. Copyright (2020) American Chemical Society.

An important aspect in the processing of cellulose in ILs is its regeneration from the liquid media. Cellulose regeneration from ILs is generally performed by adding water or other anti-solvents, causing consecutive gelation and particle formation phenomena followed by the precipitation of cellulose. The IL is then removed by filtration and further washing of cellulose with water [150]. Water is a protic solvent with a moderate value of the Kamlet–Taft parameter β (0.47) and a high value of parameter π (1.17); thus, it can interact with the hydrogen bond-accepting IL anions [184]. Zavrel et al. [168] described this process in further detail. The IL ions are dispersed in the aqueous phase by hydrogen bonding, dipolar or Coulombic forces, and water molecules form hydrodynamic shells around the IL ions, disrupting the interactions established with cellulose. The inter- and intramolecular bonds between cellulose chains are then rebuilt, enabling the polysaccharide to precipitate [168]. In this regeneration process, cellulose can be converted from cellulose I into the more stable polymorph, cellulose II. At the same time, a decrease in crystallinity can also be observed. This behavior is mainly associated with the higher freedom of movement of cellulose chains after being dissolved and regenerated. In case of cellulose regeneration after whole biomass dissolution in ILs, the disruption of cellulose interactions with other biomass components also affects the structural conformation of isolated cellulose [101]. The visual depiction of cellulose fibers after dissolution and regeneration can be found in Figure 6, which shows the complete disruption of cellulose’s fibrillar structure.

Zhang et al. [166] evaluated the efficiency of the regeneration process, after dissolving cellulose pulp fibers with [aC_1_im]Cl. The regenerated cellulose was obtained by simply diluting the IL/cellulose solution with an excess of water. The regenerated polysaccharide exhibited a DP similar to the original materials when the process was carried out at 100 °C, but it decreased slightly at higher temperatures (120–130 °C), highlighting the influence of temperature on cellulose stability along this process. Moreover, the FTIR spectra of the original and regenerated samples were similar, revealing that, apart from chain cleavage resulting in DP reduction, no reaction occurred between [aC_1_im]Cl and cellulose [166].

The problem of cellulose degradation in ILs was addressed in more detail by Xu et al. [186], who reported the addition of Na_2_HPO_3_ to avoid undesired hydrolysis of MCC during dissolution. The presence of the salt during cellulose dissolution in allyl-3-methylimidazolium methyl phosphonate ([aC_1_im][(OC_1_)HPO_2_]) revealed an interaction between Na_2_HPO_3_ and IL, inhibiting H^+^ dissociation from the cation and avoiding cellulose hydrolysis. This interaction also caused a decrease in the crystallinity of the regenerated cellulose, since the macromolecular chains exhibited difficulty in reforming the H bonds during the regeneration in the presence of salt [186]. On the other hand, when using ILs composed of cations with hydrotropic behavior, water can be inefficient to regenerate cellulose, and more hydrophobic mixtures such as water/alcohol may be required [150]. This behavior was observed by Hauru et al. [150], in which it was noted that while these type of ILs are very sensitive to water in cellulose dissolution, they tolerate it in the regeneration [150]. Overall, although cellulose regeneration from ILs has been reported in the literature [150,187], few comprehensive works have been published containing a deep study of the effect of ILs in the regeneration and structure of cellulose [173,176,180]. Grøssereid et al. [173] performed X-ray diffraction (XRD) to ascertain structural changes in cellulose after regeneration. The XRD analysis showed that all the crystallographic diffraction peaks are significantly reduced after MCC dissolution and regeneration, suggesting that the regenerated samples had a less ordered cellulose crystalline structure [173]. Kostag et al. [180] used SEM to study the morphology of the regenerated cellulose cotton samples. Depending on the IL used, in some samples, fiber morphology seemed intact, with the regenerated fibers appearing smoother than the native cotton fibers, revealing a swelling effect caused by the IL. On the other hand, when using ILs with strong interaction with cellulose, the samples revealed fibrous and non-fibrous parts [180]. 

### 2.3. Cellulose Dissolution with Deep Eutectic Solvents

The dissolution of cellulose in DESs has also been attempted in recent years. Zhang et al. [188] reported that cellulose fibers swell with cholinium acetate ([Ch][CH_3_COO]), being this an IL. However, the addition of chloride salts, such as tributylmethylammonium chloride ([N_4441_]Cl), to [Ch][CH_3_COO] forms an eutectic mixture capable of dissolving cellulose up to 6 wt%. The obtained values are similar to those achieved with some ILs [188], making this eutectic mixture a good starting point to prepare new DESs capable to dissolve cellulose. 

DESs composed of [Ch]Cl:im (3:7), [Ch]Cl:U (1:2) and [Na111]Cl:oxalic acid (1:1) are able to dissolve cotton linter pulp [189]. The imidazole-based DESs presented the highest cellulose solubility, which can be attributed to their strong hydrogen bond acceptor character. Nevertheless, a preliminary step involving cellulose activation was performed by ultrasonic-assisted treatment with saturated calcium chloride aqueous solution. The obtained results were improved (from 2.5 to 4.5 wt% cellulose solubility) when adding a co-solvent like polyethylene glycol (PEG). PEG is adsorbed to the cellulose hydrophobic domains (carbon rings of glucose) through its flexible alkoxy groups (CH_2_-CH_2_-O). This in turn allows a more effective interaction with DESs components through hydrogen bonding with the PEG hydrophilic part (hydroxyl groups). Therefore, the addition of PEG made cellulose more hydrophilic [189]. As a complementary study, allyl-functionalized DESs also demonstrated the ability to dissolve cellulose, leading to a maximum solubility of 6.48 wt% at 110 °C [190]. In both studies [189,190], the dissolution of cellulose was evidenced, since a conformation change from cellulose I to cellulose II was observed for the regenerated sample [189,190].

Abbot et al. [191] applied the DES [Ch]Cl:ZnCl_2_ for cellulose dissolution; however, only 3 wt% MCC was dissolved after applying microwave heating. This medium was then used to carry on the *O*-acetylation of cellulose with good yields [191]. Lynam et al. [192] studied the dissolution of the main fractions of lignocellulosic biomass, including cellulose, in DESs. The highest solubility of cellulose was obtained with [Ch]Cl:lactic acid (1:10), reaching ca. 3 wt% [192]. This solvent in particular has been applied successfully for delignification, leaving the cellulose structure intact [112]. More recently, Mamilla et al. [193] reported the ability of [Ch]Cl:KOH (1:4) to selectively dissolve cellulose from biomass, leaving a solid residue enriched in lignin. However, the analytical techniques used for the characterization of the obtained solid correspond only to FTIR and SEM, not allowing the determination of the chemical composition of the solid.

More recently, DESs based in carbamides (e.g., urea) have been tested for cellulose dissolution. This approach was most likely inspired by the solutions of NaOH and urea that have been conventionally applied [126]. Fu et al. [194] tested a DES composed of DBU and methylthiourea in the molar ratio of 4:1 and attained 8 wt% cellulose solubility at 40 °C under nitrogen atmosphere. Moreover, FTIR and NMR analysis as well as theoretical calculations were performed to unveil the interactions between the solvent and cellulose. The authors concluded that the dissolution of cellulose in DESs causes H bonds in the DESs to become less strong, as the HBA and HBD establish non-covalent interaction with cellulose. On the other hand, this destroys the H bonds within and between cellulose chains, making the cellulose structure loose and resulting in dissolution [194]. In another work, the dissolution of cellulose was studied at room temperature in DESs aqueous solutions containing carbamides, such as *N*-methylurea, thiourea, 1,1-dimethylurea and urea, combined with [N_4444_][OH] [195]. The molar ratio of [N_4444_][OH] and carbamides varied from 1:2 to 1:7. The best dissolution performance was obtained with [N_4444_][OH]:U (1:2) aqueous solution, which dissolved 15 wt% cellulose. The authors explained that urea can form a hydrogen bond complex with the hydroxide ion from [N_4444_][OH]. This complex then favors electrostatic interactions between hydroxide anions from [N_4444_][OH] and cellulose hydroxyl groups, favoring cellulose dissolution. Nonetheless, at a high carbamide concentration, this hydrogen bond complex may be detrimental for cellulose dissolution, since carbamides will compete for the hydrogen bond with [N_4444_][OH], decreasing the number of free hydroxide anions to interact with cellulose [195]. 

Globally, the application of ILs to dissolve cellulose has shown higher success than DESs. Recently, Abbott and Häkkinen explained the differences between the abilities of ILs and DESs for cellulose dissolution [196]. The superior solubility of cellulose in some ILs was correlated with their natural highly ordered structure, which in contact with cellulose suffers a certain degree of disorder, enabling an entropy gain. On the other hand, the lower order conformation of the DES hinders this entropy gain, resulting in lower or negligible cellulose solubility [196].

Although most of the existing DESs are unable to dissolve cellulose, they have been employed as dispersive media for the electrospinning of cellulose fibers. For instance, [Ch]Cl:urea swelled and dispersed cellulose fibers without affecting the initial cellulose I crystalline structure [197,198]. Several other studies reported the use of DESs as solvent media, either to modify and to produce new cellulosic materials [191,199] or to add new properties, such as antimicrobial activity [200]. The bulk work in this topic reveals DESs as suitable solvents for this type of application rather than cellulose dissolution.

## 3. Starch

Starch is insoluble in most conventional solvents with only few exceptions, including DMSO [86], dimethylacetamide/LiCl solutions [201] and some amines and their derivatives [202]. Rather than enabling dissolution, thermoplasticization is an important industrial method for starch processing, in which the starch granules become amorphous by the presence of a polar plasticizer at high temperatures. The material then becomes more elastic and forms the homogeneous and transparent thermoplastic starch (TPS) [202]. The most common starch plasticizers are water and glycerol, although polyols, amines, amides and polycarboxilic acids are also used. Nevertheless, these plasticizers still present some limitations, such as the recrystallization of starch resulting in an undesired increase in the stiffness and brittleness of the material [113]. For these reasons, research for new solvents and plasticizers to produce new starch-based materials is ongoing, where ILs and DESs can stand as good alternatives.

### 3.1. Starch Dissolution and Plasticization with Ionic Liquids

Similar to cellulose, ILs have been applied in starch dissolution successfully (Table 6). Likewise, the dissolution mechanism is also related to the IL ability to break the hydrogen bonding network present in the starch semi-crystalline structure. ILs containing Cl^−^ or [CH_3_COO]^−^ anions and imidazolium cations have been identified as good solvents for starch [203]. Regarding the cation, a smaller size leads to higher starch dissolution, e.g., [C_2_C_1_im]^+^ vs. [C_6_C_1_im]^+^ [204]. 

**Figure 7:**
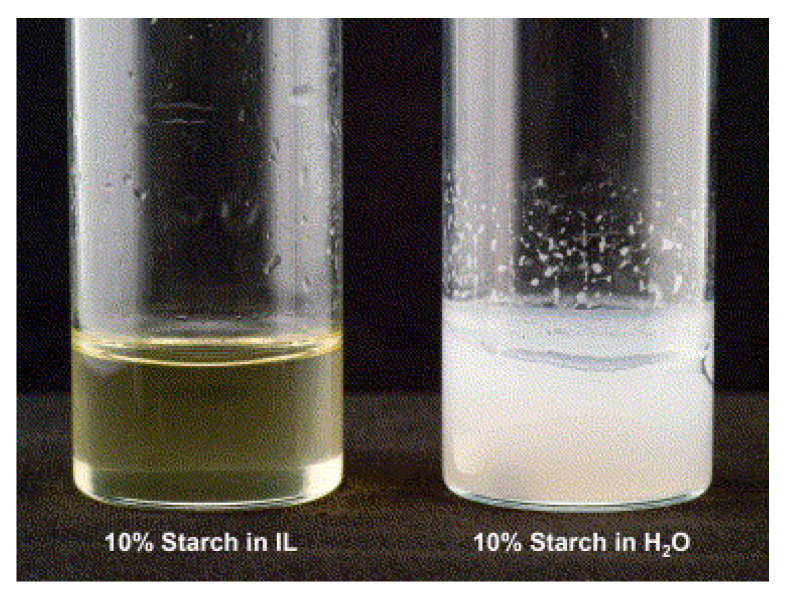
Picture demonstrating total solubilization of starch (wt.%) in 1-butyl-3-methylimidazolium dicyanamide and partial solubilization in water. Reprinted from [52], with permission from Elsevier.

Both [C_4_C_1_im]Cl and [aC_1_im]Cl have shown the ability to dissolve starch (10–20 wt%) [205,206,207] and the same applies for [C_4_C_1_im][N(CN)_2_] that reached 10 wt% starch solubility at 90 °C. On the opposite, starch was found to be insoluble in 1-methylimidazolium tetrafluoroborate ([C_4_C_1_im][BF_4_]), revealing the importance of the anion selection (Figure 7) [52]. Moreover, starch dissolution is highly dependent on the starch composition (amylose/amylopectin ratio) and on the temperature [52].

Another interesting strategy in starch processing is the gelatinization/plasticization process. Starch can undergo gelatinization when suspended and heated in water. This phenomenon is an order–disorder transition in which the starch granules swell, and the amylose is progressively leached from the granules disrupting the structure. Although some granules are soluble in water, others are not and this partial solubility is a problem, especially in the production of materials [51]. On the other hand, no gelatinization takes place during starch dissolution in pure ILs, due to starch disruption in the IL media. This means that an exothermic transition occurs and starch granules do not disorganize and swell [51]. However, the dissolution of this type of polysaccharide can follow different trends depending on the IL concentration. At low IL concentrations, the gelatinization process is favored, while depolymerization may take place at higher IL concentrations due to the hydrolysis of glycosidic bonds, even at low temperatures, resulting in lower-molecular weight structures and jeopardizing starch applications as materials [51,203].

It has been noted that starch from corn, rice, wheat and barley is easier to gelatinize and dissolve than starch from waxy corn and potato. This behavior is directly correlated with the size of the starch granules but also with the higher amylopectin content of the latter two types [53]. It is important to highlight that depolymerization of amylose and amylopectin may occur during this process, especially when working at high temperatures and high IL concentrations [203]. 

Furthermore, while ILs with cations such as [C_2_C_1_im]^+^ have a good effect in starch solubility, the choice of the cation must be well thought out. For instance, the presence of Cl^−^ promotes hydrolysis of the glycosidic bonds and subsequent depolymerization of starch [51,203]. On the other hand, [C_2_C_1_im][CH_3_COO] aqueous solutions in the proportion 0.15/1 (mol_IL_/mol_water_) were found to dissolve a maximum of 10 wt% starch at room temperature, without any degradation of starch [203]. Although the solubility is lower than that obtained with chloride-based ILs, [C_2_C_1_im] [CH_3_COO] could be a good alternative to apply at moderate temperatures. Furthermore, the relatively high solubility of starch in [C_2_C_1_im][CH_3_COO] aqueous solutions indicates that water does not act as an anti-solvent in these systems, which is the opposite to that reported for cellulose dissolution [203].

Co-solvents may also be applied with ILs to decrease viscosity, to reduce starch degradation and to increase starch solubility [58]. For starch, the addition of water exhibits a completely opposite effect when compared to cellulose, since it helps solubilization. This behavior was reported in the work of Sciarini et al. [51], where the ILs acted as stabilizing salts for gelatinization in water. A careful selection of the IL/water ratio was reported as the key to obtain a good dissolution of starch with minimal losses [51,208].

Wang and co-workers have published two works [209,210] in which they studied the effect of water in starch solubilization and its relationship with the IL cation and anion. It was observed that the IL cation influence on starch solubility can be inverted with different water molar ratios; for instance, at H_2_O:IL ratios of 10:1 and 10:2, starch solubility followed the order [C_4_C_1_im]Cl > [C_3_C_1_im]Cl > [C_2_C_1_im]Cl, but at higher amounts of IL (e.g., 10:5), ILs with smaller cations were more effective in dissolving starch. These results were explained by the higher physical interaction of a larger cation, favoring the IL interaction with starch, and also by the decrease in solvent viscosity when water is added. The last reason is especially relevant for larger cations that produce more viscous ILs when the water content is low [209]. Furthermore, a similar trend was observed when screening the IL anion effect [210]. The disruption extent of starch structures was ordered as follows: [C_2_C_1_im][CH_3_COO] > [C_2_C_1_im][HCOO] > [C_2_C_1_im]Cl at water/IL ratios of 10:1 and 5:1, but the opposite was observed with a lower amount of water (2:1). This means that [C_2_C_1_im]Cl increased its ability to disrupt the starch structure with a higher IL content. In this work, authors mentioned that the hydrogen bonding acceptor capacity of the IL anions plays a major role in starch dissolution at a low IL content, whereas the viscosity of the water/IL mixtures exhibit a major impact on starch dissolution at a high IL content [210]. Both studies revealed that cation, anion and water contents are key factors to tailor starch solubility in ILs as well as the starch phase transitions and gelatinization process. At a high water content, the interaction between starch and water dominates, resulting in an endothermic transition of starch (gelatinization). On the opposite, the interaction between starch and IL prevails at a low water content, leading to an exothermic transition of starch (dissolution) [211].

Organic co-solvents such as DMSO or methanol can be applied to increase starch solubility. For example, a low molar fraction of DMSO (<0.4) can weakly associate with the IL cation, competing with the strong cation–anion interaction in pure IL and thus increase starch solubility [212]. This results in the anomalous diffusion (bulky cation moving faster than the anion in pure IL) being reduced. Above 0.6 molar fraction of DMSO, the anion diffuses in a “normal” way, that is, faster than the cation [212]. This discovery can explain the role of DMSO both in starch and cellulose dissolution as referred to above [212]. This effect was also noticed by Gao et al. [213], who demonstrated a decrease either in temperature or time needed to solubilize 10 wt% of starch when DMSO was added to [C_2_C_1_im]Cl (1:1 in wt). However, it was noted that a complete destruction of the structure of starch granules may occur at high DMSO contents [213].

Shen et al. [214] revealed that methanol can accelerate the dissolution of corn starch in ILs by swelling the outer layer of starch and penetrating into the granules. Methanol can thus be more efficient to swell hydrophobic molecules in the granules’ outer layer (phospholipids). In this case, methanol was added to a mixture of [(C_1_)_2_IC_1_][(C_1_O)HPO_2_]/water (7/3) at a ratio of 8/2 (*w/w*), causing an acceleration in the starch dissolution rate by decreasing the activation energy required for dissolution to occur [214]. It was also demonstrated that at this specific ratio, both exothermic and endothermic transitions occurred, meaning that both gelatinization and dissolution took place. However, gelatinization was the main process at higher contents of methanol [214]. 

To promote faster starch dissolution in ILs, microwave-assisted heating was studied. Lajunen and co-workers [204] obtained a maximum of 10 wt% starch dissolution with dialkylimidazolium-based ILs with halide anions. The process was handled at 80 °C within timeframes between 0.33 and 3 h. Researchers also benchmarked with conventional heating and confirmed the suitability of microwave-assisted processes to decrease the dissolution time [204]. Furthermore, this study allowed a comparison between different cations, revealing that cations with longer alkyl chains resulted in slower dissolution times, which is related to their steric hindrance [204].

### 3.2. Starch Dissolution and Plasticization with Deep Eutectic Solvents

Regarding the application of DESs in starch dissolution, several works have been disclosed [113,184,217,218,219,220] (Table 7). Carboxylic-acid-based DESs have been tested, and good results were obtained with oxalic or malic acids as part of the solvent [217,219,220]. The DESs’ performance for starch solubility is as follows: glycine:MA (1:1) > [Ch]Cl:MA(1:1) > proline:MA(3:1) > [Ch]Cl:oxalic acid (1:1), with 7.65 wt% being the highest starch solubility attained [217]. The DES 1-methyl-2-oxopyrrolidinium chloride:oxalic acid (1:1) led to 10 wt% starch solubility at room temperature [219]. However, it is unclear if starch depolymerization occurs during the dissolution process, especially when considering the acidity of oxalic acid. The same problem can be associated with other DESs containing an excess molar ratio of organic acid (e.g., histidine/alanine:LA (1:9) or [Ch]Cl:LA (1:10)) that have been tested in starch dissolution (Table 7) [217,220]. On the other hand, DESs possessing citric and succinic acids cause starch darkening and degradation, with low solubility values reported [113]. Furthermore, these DESs disable the plasticization of starch, a common trend in other applied DESs [113]. Other DESs combinations have been applied to starch solubilization, especially those composed of [Ch]Cl as the HBA. For instance, [Ch]Cl:ZnCl_2_ was able to solubilize 5–10 wt% starch at 100 °C [52], while [Ch]Cl:im dissolved 20 wt% of starch at the same temperature, plasticizing it at lower temperatures (77 °C) [113]. Furthermore, sugar-based DESs, including [Ch]Cl:glucose (1:1) and glucose:LA (1:5), were also used in starch dissolution [218,220], but low solubilities were found.

Similar to ILs, the ability of DESs to dissolve starch is attributed to the strong hydrogen bond network formed between the solvent and the polysaccharide hydroxyl groups. These interactions were further confirmed by Dai et al. [220] through nuclear magnetic resonance (NMR). It has been verified that water may affect starch solubility in DESs. Mąka and co-workers followed starch dissolution in [Ch]Cl:im through differential scanning calorimetry (DSC) and noticed that the exothermic peak was more pronounced in dry then hydrated starch [113], which was associated with the swelling of starch granules in the presence of water. Moreover, [Ch]Cl:im is solid at room temperature and water might decrease the crystallization and viscosity of the solvent [113]. On the other hand, gelatinization may also occur at low DESs contents since endothermic peaks were displayed in the DSC analysis. Therefore, starch dissolution in DESs seems to follow the same trend displayed in ILs, i.e., gelatinization is enabled at low DESs concentrations, while starch dissolution is favored at high DESs concentrations [113].

In summary, only few works have focused so far on starch dissolution with DESs. A primary focus has been given towards the study of the plasticization of starch with these solvents to improve the mechanical properties of thermoplastic polymers [222,223]. However, an increasing knowledge behind starch dissolution mechanisms in DESs is expected to come in the near future. The bulk of work on starch dissolution is much more extensive with ILs and the dissolution mechanisms have been comprehensively addressed [51,52,208]. ILs have also been used in the plasticization process of starch (e.g., [C_4_C_1_im]Cl and [aC_1_im]Cl), and as explained above, this phenomenon can be easily tailored by playing with the water and IL ratio. Similar conclusions can be translated to DESs, particularly when sharing similar chemical structures to ILs in their composition. To the best of our knowledge, no further studies applying ILs or DESs in the selective extraction of starch from biomass sources have been performed so far and this is a line of research to be explored.

## 4. Hemicelluloses

Hemicelluloses are a diverse group of polysaccharides, which implies a wide range of extraction methodologies and conditions that are dependent on their origin and structure. In general, branched hemicellulose structures are more difficult to dissolve; additionally, the covalent linkages with lignin and cell wall proteins also hinder their solubility. On the other hand, acetylated structures, including xyloglucans, galactomannans and glucomannans are more water-soluble [39]. Furthermore, the solubility of xylans composed of side chains containing arabinose is dependent on the content and distribution of arabinose units [224,225]. 

The standard approaches often used to dissolve hemicelluloses comprise the employment of alkali aqueous solutions [128] or DMSO. In the case of DMSO, water can be added as a co-solvent to increase the solubility of low-branched xylans [226]. At the industrial scale, the most common approaches are hydrothermal, acid and alkaline treatments [227]. However, these methodologies result in the depolymerization of hemicelluloses and their degradation into by-products (either unwanted or value-added) as a consequence of harsh acidic or alkaline conditions coupled with high temperatures [227].

### 4.1. Hemicelluloses Dissolution and Extraction with Ionic Liquids

The use of ILs to dissolve and extract hemicelluloses has been demonstrated as an alternative solution to conventional processes. In the dissolution context, Yuan et al. [228] studied the dissolution of bamboo hemicelluloses in three ILs, namely [C_4_C_1_im]Cl, [C_4_C_1_im]Br and [C_4_C_1_im]I, at temperatures ranging from 80 to 150 °C. The authors noticed that the solubilities followed the order: [C_4_C_1_im]Cl > [C_4_C_1_im]Br > [C_4_C_1_im]I, which is in accordance with these ILs’ ability to dissolve cellulose [229]. The authors studied the interactions between ILs and hemicelluloses through NMR and concluded that the anion basicity is key for dissolution, while the cation interaction was not so significant, again similarly to the process behind cellulose solubility. The highest solubility achieved was 18.8 wt% at 120 °C, using [C_4_C_1_im]Cl. Authors also performed the recovery and characterization of hemicelluloses, revealing that their structure was practically maintained during the process [228]. 

The previous study [228] and comparison to cellulose data evidence a major limitation with ILs in what partakes to hemicellulose dissolution or extraction—a lack of selectivity. In fact, ILs capable to dissolve cellulose instead of lignin [230], as well as ILs able to dissolve both hemicelluloses and lignin rather than cellulose, have been reported [231]. However, a scarce number of studies have addressed the use of ILs to selectively dissolve hemicelluloses [232]. Generally, ILs that can dissolve cellulose are able to dissolve hemicelluloses [233]. Therefore, rather than approaching a selective extraction, whole dissolution of raw materials in ILs is usually attempted in the literature. Afterwards, biomass components are fractionated and isolated by the addition of selective anti-solvents to the IL/biomass mixture [5,181,182,234,235]. In this context, Cheng et al. [236] published a study upon sugarcane bagasse pulping and fractionation using ILs, focusing on the separation of the three main lignocellulosic fractions. An evaluation of the solubility of model compounds (xylan, MCC and kraft lignin) was conducted in the first place using cholinium acetate ([Ch][CH_3_COO]) and [Ch][CH_3_COO] in a water mixture (5:1 (w:w)) followed by pulping trials. The solubility assays revealed a maximum solubility of xylans (13 wt%) with both neat IL and its aqueous solution. However, temperatures required for the dissolution in the IL aqueous solution were lower and thus preferred. Furthermore, the solubility assays demonstrated low solubility of cellulose in this IL (<0.50 wt%), making it ideal for a selective extraction of hemicelluloses from biomass [236]. Three different fractionation methodologies were evaluated and a maximum hemicellulose yield of 22.5 wt% was achieved. Hemicelluloses were obtained by precipitation with ethanol directly from the biomass/IL mixture, while lignin was recovered by precipitation with water. IL recycling was also taken into consideration, with a 95 wt% recovery reported. The recovered IL was used in one more extraction cycle without losing its extraction efficiency [236]. A similar approach was successfully achieved for wheat straw fractionation using the same IL, with similar results [234]. Li et al. [232] also attempted hemicellulose extraction from *Eucalyptus* species of wood, namely from *E. grandis* and *E. urophylla*, by first pre-treating biomass with [C_4_C_1_im]Cl and [C_4_C_1_im][CH_3_COO] (5 wt% biomass loading/120 °C), followed by diluted aqueous NaOH treatment. A maximum hemicellulose yield of 22.42 wt% was obtained using [C_4_C_1_im] [CH_3_COO] in the first step, while 18.22% yield was attained without this pre-treatment. Furthermore, the pre-treatment with IL resulted in hemicelluloses with higher M_w_ than those obtained in the absence of pre-treatment [231]. Comparing the works of Cheng et al. [236] and Li et al. [232], the obtained hemicellulose yields are very similar, although the choice for cholinium acetate seems to be promising for hemicellulose extraction as no additional alkaline extraction is needed. Furthermore, the first study [236] further confirms the advantage of performing preliminary studies with model compounds to give some insights about solubility mechanisms and adequate conditions for the extraction process.

Since the key to dissolve cellulose with ILs is based on the high basicity of the anion, the challenging selective extraction of hemicelluloses from biomass might be done by reducing it. In this line, Froschauer et al. [237] replaced one of the oxygen atoms in the phosphate anion of 1-butyl-3-methylimidazolium dimethyl phosphate ([C_4_C_1_im](C_1_)_2_PO_3_) by sulfur or selenium to decrease its hydrogen bond basicity [238]. Indeed, this structural change in the IL allowed the dissolution of xylan without affecting cellulose. However, a similar assessment was not performed in the evaluation of IL selectivity for lignin versus xylan dissolution. Furthermore, the introduction of sulfur or selenium has a negative impact on IL toxicity that could undermine a possible industrial application [237]. In another work, instead of chemically modifying the IL, Pang et al. [231] tuned the solvent properties by using co-solvents. For instance, the addition of LiCl aqueous solution to [C_1_C_4_im]Cl (a good cellulose solvent) allowed dissolving only lignin and hemicelluloses [231].

### 4.2. Hemicellulose Dissolution and Extraction with Deep Eutectic Solvents

**Figure 8:**
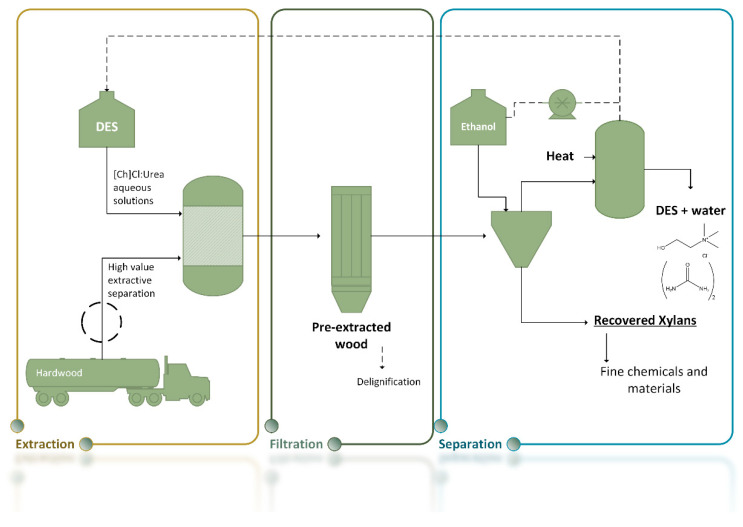
Schematic representation depicting an integrated process within biorefinery using [Ch]Cl:U aqueous solutions to extract xylans from hardwood (adapted from [238]).

Similar approaches regarding hemicellulose dissolution and extraction from biomass to those reported above for ILs have been proposed with DESs. Lynam et al. [192] studied the dissolution of the main fractions of lignocellulosic biomass, including hemicelluloses, in DESs. The highest solubility of hemicelluloses was obtained with [Ch]Cl:LA (1:10), reaching up to 5 wt% [192]. However, solubility assays were performed by visual observation, in which a clear solution indicated dissolution; yet, no evidence about the integrity of the dissolved hemicelluloses was shown. In addition to neat DESs, Morais et al. [238] studied several aqueous solutions of DESs to dissolve xylan, with further extraction studies of xylan from wood. The [Ch]Cl:U(1:2, at 50 wt%) aqueous solution revealed to dissolve xylan up to 328.23 g.L^−1^ at 80 °C. This study also revealed that an inverse relationship between DES concentration and temperature governed the dissolution efficiency. Moreover, the use of water is a clear advantage to the use of pure DESs, allowing to tailor the process through manipulating its amount. As proof of concept, the extraction of xylan from *E. globulus* wood gave a maximum yield of 14.8 wt%. This study envisioned an integrated approach with DESs aqueous solutions, contemplating solvent recovery and recycling, as summarized in Figure 8 [238]. These two works are, up to date, the only reports addressing hemicelluloses dissolution and selective extraction from biomass with DESs, indicating that this field is still in its infancy and promising results can be obtained by a proper DESs tailoring.

Hemicellulose dissolution and selective extraction from biomass is still regarded as a secondary approach in lignocellulosic biomass processing when compared to cellulose valorization. Even though the application of ILs in hemicelluloses valorization has been scarcely approached, there are already some ILs that display the capability to selectively dissolve hemicelluloses. Although chemical modifications to ILs’ structure have been attempted to make them more selective towards this polysaccharide, only few studies have shown success. The use of IL aqueous solutions could also be a good alternative to selectively extract hemicellulose, since as mentioned above, water generally hinders cellulose dissolution. Moreover, the use of aqueous solutions would also result in lower solvent viscosity, a lower energetic input and a decrease in cost. On the other hand, the use of DESs for the same purpose is still in its infancy and there is a lack of knowledge. Nonetheless, the use of aqueous solutions has already been attempted with good results. However, it is important to note that DESs’ recyclability was already shown after hemicellulose extraction, demonstrating their potential as polysaccharides-upgrading solvents.

## 5. Pectin

Pectins are water soluble, but their dissolution rate varies widely with their degrees of polymerization and branching. Temperature, pH and ionic strength of the solution are of high importance to achieve an improved pectin dissolution rate. The calcium content in water is also relevant, once high water hardness is translated into inefficient pectin dissolution rates [82]. In addition, the main obstacles in dissolving these polysaccharides are related to their complex chemical structure and close association with the cell wall polymers [239]. Pectin extraction is usually made by employing dilute aqueous solutions of mineral acids (hydrochloric, sulfuric and phosphoric), at pH 1.5–3.0 and at mild to high temperatures. The process can take up to two hours and may cause unwanted pectin degradation as a consequence of the acidity and high temperatures applied. Due to environmental concerns, more sustainable technologies have been developed for pectin extraction, including UAE, MAE and accelerated solvent extraction (ASE) [66]. These technologies have been applied with water or aqueous diluted organic acid solutions as extraction solvents [240,241,242,243]. Although a considerable decrease in the extraction time is achieved with these methods, enabling less pectin degradation, there is still a lack of selectivity in the extraction. On the other hand, technologies based on enzymatic cleavage have been attempted to increase pectin extraction selectivity, but limitations upon the enzyme specificity and also the scale-up costs are disadvantages [244]. The use of ILs and DESs may be a way to increase pectin selectivity in these processes, specifically when coupled with the abovementioned technologies, due to their “designer solvent” characteristic. 

### 5.1. Pectin Extraction with Ionic Liquids

Contrary to the other polysaccharides discussed in appraisal, to the best of our knowledge, there are no reports on pectin solubility in ILs. Only a single study reported the ability of [C_4_C_1_im]Cl to dissolve pure polygalacturonic acid (5.3 wt% at 140 °C) as a model component of the pectin backbone [245]. The mechanism of pectin dissolution in ILs is expected to be similar to that described for cellulose, in which hydrogen bonds between the IL anion and the OH groups of the pectin polysaccharides are established. However, another factor was specifically associated to [C_4_C_1_im]Cl, which, according to the authors, liberates hydrochloric acid at high temperatures leading to the formation of poly-galacturonic acid salt with the imidazolium cation as the counterion, thus increasing the solubility of the polysaccharide [245].

Regarding pectin extraction from biomass with ILs, there are, however, some (few) works published (Table 8) [244,246]. Wang et al. [246] applied several bio-based ILs comprising the cholinium cation and different amino acid-derived anions, namely ([Ch][Ala], [Ch][Ser], [Ch][Cys], [Ch][Pro], [Ch][Asp], [Ch][Val], [Ch][Leu] and [Ch][Phe]), to extract pectin and flavonoids from ponkan peels. After the extraction step, both extracted components were separated by an aqueous biphasic system, achieving a pectin yield of 13.42 wt% [246]. However, pectin extraction with ILs could be coupled with MAE and/or UAE to increase the extraction efficiency, as shown by Liu et al. and Guolin et al. [244,247]. Liu et al. [244] applied 3-methyl-1-(4-sulfonylbutyl)imidazolium hydrogensulfate ([SO_3_HC_4_C_1_im][HSO_4_]) as an extraction solvent of pectins from pomelo peels coupled with both ultrasound- and microwave-assisted extraction (UMAE). A maximum pectin yield of 328.64 ± 4.19 mg/g was obtained with this technique, which was significantly higher than extraction yields achieved with conventional heating (194.24 ± 5.71 mg/g) or UMAE (269.31 ± 5.71 mg/g), both using aqueous hydrochloric acid. Furthermore, the obtained pectins had no significant differences in carbohydrate content and esterification degree in comparison to the native polysaccharide [244]. Additionally, the IL was shown to be recycled five times by vacuum distillation with minimal yield losses (ca. 10%). MAE was also coupled with ILs to extract pectins from pomelo peels [247]. ILs, such as [C_4_C_1_im]Cl, [C_4_C_1_im]Br, [C_4_C_1_im][BF_4_], [C_2_C_1_im]Cl, [C_2_C_1_im]Br and [C_2_C_1_im][BF_4_], were applied and the extraction time, temperature and solid/liquid ratio were optimized. The best pectin yield (24.68 wt%) was obtained with [C_4_C_1_im]Cl at 88 °C for 9.6 min with a liquid–solid ratio of 22.7 mL g^−1^. Once more, the yield attained with conventional methodologies (12.73 wt%) was surpassed with much lower extraction times (9.6 min vs. 2 h) [247].

### 5.2. Pectin Extraction with Deep Eutectic Solvents

**Figure 9:**
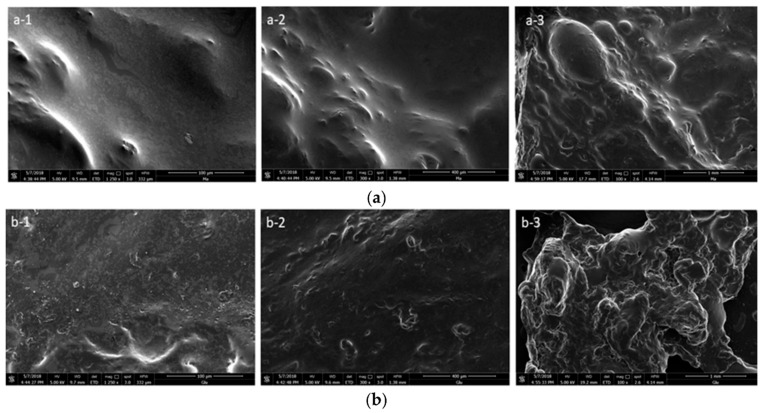
(**a**) Electron images of pectin extracted by [Ch]Cl–malonic acid; (**a-1**) (1250×), (**a-2**) (300×) (**a-3**) (100×) and (**b**) electron images of pectin extracted by [Ch]Cl:glucose–water; (**b-1**) (1250×), (**b-2**) (300×) (**b-3**) (100×). Reproduced from [249].

As with ILs, solubility studies of pectin with DESs are not available in the literature, and the application of DESs in pectin extraction has been barely studied (Table 9). Pectin extraction from pomelo peels was attempted using aqueous solutions of DESs composed of lactic acidic and glucose or glycine [248]. However, the pectin yields did not surpass those obtained with aqueous solution of citric acid (39.72 wt%), which is already being employed as an alternative to mineral acids. The best result for DESs (23.04 wt%) was attained with glucose/LA/water in a molar ratio of 6:1:6. An additional work focused on using DESs with a sonoreactor to extract pectins, also from pomelo peels [249]. [Ch]Cl-based DESs based on sugars (e.g., glucose and fructose), carboxylic acids (e.g., oxalic, malonic and citric acids) and polyols (e.g., glycerol) were used and compared with the aqueous solutions of individual compounds. Moreover, some DESs were tested with and without water. The operating conditions were fixed at 60 °C and 120 min of extraction time. The best extraction yields were attained with [Ch]Cl:malonic acid (1:1) and [Ch]Cl:glucose:H_2_O (5:2:5), whereas the operating conditions were optimized through a response surface methodology. Approximately 94 wt% pectin yield and 52% degree of esterification (DE) were achieved using [Ch]Cl:malonic acid (1:1), while [Ch]Cl:glucose:H_2_O (5:2:5) reached 87 wt% pectin yield and 54% DE. However, the authors noted differences in the morphology of the extracted pectin. The acid-based pectin was easy to filter, compact and gelatinous, obtaining a smooth and shiny morphology, while the glucose-based pectin was difficult to filter with a loose structure and a rough surface (Figure 9). Therefore, due to both yield and morphology, the acidic extraction of pectin is preferred [249].

## 6. Chitin

Chitin is highly insoluble in water and treatment with strong aqueous HCl solutions is in general applied to overcome this issue. The acid treatment allows inter-crystalline swelling and switches the initial β-chitin to an α-chitin conformation. However, this also leads to chain scission and decrystallization of the polysaccharide. After washing with water, partial recrystallization occurs and a mixture of α-chitin and β-chitin fibrils is formed [250]. Furthermore, the acetyl group distribution also has an influence on chitin solubility. Chitin typically presents a DA near 0.90 [71], whereas the acetyl favors chitin dissolution [251]. In general, cellulose solvents such as DMAc with LiCl are also able to solubilize chitin [252] as well as strong polar protic solvents, including trichloroacetic acid (TCA) and dichloroacetic acid (DCA) mixed with dichloromethane (DCM) [71]. Nevertheless, while chitin is insoluble in most organic solvents and water, its derivative chitosan is soluble in aqueous systems at dilute acidic conditions (e.g., pH < 6.0). At low pH, the amino groups become protonated (chitosan pKa ≈ 6.3), giving to chitosan a positive charge that acts as a water-soluble poly-electrolyte [71]. The most commonly used solvent for chitosan dissolution is 1% aqueous acetic acid [253]. Other aqueous acid solutions, incorporating formic, hydrochloric, nitric and lactic acids, also dissolve chitosan [13,71,254]. Many of the solvents described and commonly applied are toxic, corrosive, nondegradable or mutagenic and lead to the generation of waste that is difficult to process [255]. These disadvantages move forward the search for greener and sustainable solvents capable to dissolve and extract this polysaccharide from raw materials.

### 6.1. Chitin and Chitosan Dissolution and Chitin Extraction with Ionic Liquids

Several works have already reported the dissolution of both chitin and chitosan in ILs, as summarized in Table 10 [77,255,256,257,258,259,260]. Chitin dissolution is complex and depends not only on the strong hydrogen bond acceptor ability of the IL anion and its interaction with the cation, but also on the chitin type and degrees of acetylation and crystallinity [261]. Regarding chitin, the highest solubility was reported for [C_2_C_1_im][CH_3_COO], being approximately 20 wt% with microwave irradiation [258]. 

Interestingly, chitin was insoluble in [aC_1_im]Cl, while up to 10 wt% chitin solubility was achieved with 1-allyl-3-methylimidazolium bromide ([aC_1_im]Br). In the presence of the [aC_1_im] cation, Br^−^ ion was suggested to play an important role in chitin dissolution, rather than its Cl^−^counterpart. However, by replacing [aC1im] with [C_4_C_1_im] and using the same anions, the highest solubility was observed with the Cl-based IL [257]. On the other hand, when using the [C_2_C_1_im] cation, the best solubility was achieved with the [CH_3_COO]^−^ anion (20 wt%) [258]. This suggests that the interaction between the IL cation and anion is crucial in the chitin dissolution process. It is important to note that the IL comprising the [aC_1_im]^+^ cation seems to be an exception, because other cations perform better for chitin dissolution when coupled with the Cl^−^ and [CH_3_COO]^−^ anions (Table 8). As with other polysaccharides, combining ILs with other alternative technologies is quite advantageous to achieve better dissolution results. In the case of chitin dissolution in ILs, microwave induction has been used successfully. In the work disclosed by Qin et al. [258], a remarkable dissolution of 20 wt% chitin was achieved using [C_2_C_1_im][CH_3_COO] by a conventional heating method after 19 h at 100 °C, while the same dissolution yield was attained with microwave full power with pulses of 3 s in only 2 min [258].

Chitosan dissolution was attempted with [C_2_C_1_im][CH_3_COO], [C_4_C_1_im][CH_3_COO], [C_4_C_1_im]Cl and [aC_1_im]Cl [256]. The solubility data allowed to order the ILs’ performance in the following trend: [C_2_C_1_im][CH_3_COO] < [C_4_C_1_im][CH_3_COO] < [aC_1_im]Cl < [C_4_C_1_im]Cl, with [C_2_C_1_im][CH_3_COO] being able to solubilize up to 15 wt% [94]. It is important to note that since chitosan is readily soluble in mild acid conditions, the dissolution with ILs can only be justifiable when high chitosan concentrations are required in the solvent. Analogous to cellulose, the higher the hydrogen bond-accepting ability of the IL anion, the higher the chitin solubility. Therefore, [CH_3_COO]^−^ is a better choice than Cl^−^ since it presents higher basicity to form hydrogen bonds with both the -OH and -NH_2_ groups of chitosan. Since the -NH_2_ groups are more prone to form hydrogen bonds than the -OH groups, the hydrogen bonding network between chitosan and ILs is different and more complex than in the case of cellulose [94]. Regarding the influence of the IL cation on chitosan dissolution, Xiao et al. [257] proposed that the IL cation interacts with the oxygen atom in the same groups, disrupting the intrinsic hydrogen bonds of chitosan [257]. This interaction was confirmed by ^13^C-NMR studies in which the carboxyl group of the IL anion interacts with the hydrogen atoms of –OH and –NH_2_, while C2 from the cation interacts with the O and N atoms of the same groups [262]. 

Regarding the extraction of chitin from food waste (e.g., crabs and shrimp shells) using ILs, most of the works reported in the literature focused on the complete dissolution of biomass followed by the selective precipitation of chitin and separation of contaminants (e.g., proteins and mineral fraction). The MAE of chitin from crustacean shells with [C_2_C_1_im][CH_3_COO] showed a maximum of 94 wt% chitin yield [258]. In this work, a comparison between the Cl^−^ and [CH_3_COO]^−^ anions was also carried out, revealing higher efficiency of [CH_3_COO]^−^ in chitin extraction due to its higher hydrogen bond basicity [258]. Due to these remarkable results, Rogers and co-workers later scaled-up the process and founded the company 525 Solutions [131] that focuses on chitin extraction from shellfish waste, being this one of the examples where ILs are actually applied at an industrial scale. 

[C_4_C_1_im][CH_3_COO] is capable to dissolve both α and β conformations of chitin, achieving solubility values between 3 and 7 wt% [259]. It should be highlighted that this work was based on the complete dissolution of raw crustacean shells in ILs, followed by chitin precipitation and recovery through the addition of an anti-solvent (methanol). Nevertheless, this method faces a big challenge in overcoming the separation of a high mineral content of such materials that undermine the purity of extracted chitin. Moreover, the use of the toxic methanol as an anti-solvent is not ideal [259].

Following a similar rationale, Setoguchi et al. [263] used [aC_1_im]Br to dissolve chitin from crab shells at 80–120 °C and subsequently citric acid was added as a coagulant and anti-solvent to precipitate the polymer and to simultaneously demineralize the sample. This work revealed that higher temperatures increase the chitin yields (12.6 wt%), although extracted chitin presented a lower molecular weight than that obtained at lower temperatures [263].

In addition to aprotic ILs, protic ILs have been investigated as well to extract chitin. Ammonium-based ILs, namely dimethylbutylammonium acetate ([N_114_][CH_3_COO]), diisopropylethylammonium acetate ([N_332_][CH_3_COO]) and diisopropylethylammonium propanoate ([N_332_][CH_3_(CH_2_)_2_COO]) were able to selectively extract chitin from dried ground shrimp shells [264]. Similarly, citric acid was used as a coagulant of chitin. After 36 h of extraction at 110 °C with 6 wt% of biomass in the IL, a maximum yield of 14.8 wt% chitin was achieved using [N_332_][CH_3_COO]. A decrease in the polysaccharide molecular weight was also observed with higher temperatures of extraction [255], being in agreement with the work of Setoguchi et al. [263] using aprotic ILs. 

The main limitation of ILs in chitin dissolution comprises the lack of selectivity, but also the recovery of the polysaccharide from the liquid media. The conjugation of the IL as a solvent with other green technologies could be a solution. For instance, CO_2_ can be employed as a drying agent to remove residual [C_4_C_1_im][HCOO] from the regenerated chitin [265]. Regarding chitosan, Mu and co-workers [266] applied compressed CO_2_ as a way to recover chitosan from [C_4_C_1_im][HCOO]. The precipitation of chitosan was induced by CO_2_, leading to the creation of a gas-expanded liquid. This technology is also a good approach for IL recovery and recycling.

### 6.2. Chitin and Chitosan Dissolution and Chitin Extraction in Deep Eutectic Solvents

Chitin dissolution in DESs has been attempted with higher success than what has been reported for cellulose dissolution. [Ch]Cl:U (1:2), [Ch]Br:U (1:2), [ClCh]Cl:Ur (1:2) and Betaine hydrochloride:U (1:4) are able to dissolve α-chitin in a vacuum atmosphere. The highest chitin solubility was obtained with [Ch]Cl:U(1:2) (8.0 wt%) after heating at 100 °C for 10 h [278]. In this study, microwave and ultrasound were also applied and chitin solubility was improved. Other DESs composed of an IL as the HBA component have also been used for chitin dissolution. That is the case of tris(2-hydroxyethyl) methylammonium mixed with ethylenediamine (EDA) and anions such as [MeSO_3_]^−^, [CF_3_SO_3_]^−^, [Tf_2_N]^−^ and [CH_3_COO]^−^, which have been reported as being able to dissolve chitin. However, solubility values were not disclosed by the authors [279].

**Figure 10:**
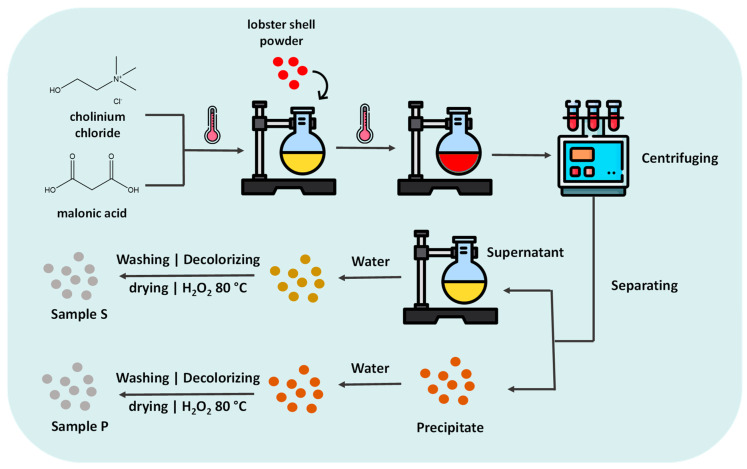
Schematic representation depicting the methodology for chitin extraction from lobster shells using [Ch]Cl:Malonic acid. Adapted from [280].

Extraction of chitin from biomass has also been approached using DESs. Zhu et al. [280] extracted chitin from lobster shells using [Ch]Cl:thiourea, [Ch]Cl:glycerol and [Ch]Cl:malonic acid. The extraction was carried out at 50–110 °C and from 2 to 6 h, depending on the examined DES, while mass ratios of lobster shells/DES between 5 and 20 wt% were used. Among the examined DESs, [Ch]Cl:malonic acid presented the best chitin extraction yield at 50 °C for 2 h and using 7 wt% ratio of lobster shells/DES. A summary of the methodology adopted in this work is summarized in Figure 10. In the aforementioned conditions, two fractions of chitins were obtained: one from the washing of the precipitate (MA-P) and another from the washing and centrifuging of the extraction supernatant (MA-S). The XRD analysis of these fractions showed both to possess a stable α-chitin form, although with different crystallinity indexes: 67.2 ± 0.9% for sample MA-S and 80.6 ± 0.8% for sample MA-P, corresponding to 4.44% and 16.19% yield, respectively. These results show that [Ch]Cl:malonic acid is able to disrupt intra- and intermolecular hydrogen bonds between chitin chains, enabling to change the crystalline structure [280]. Furthermore, [Ch]Cl:malonic acid was able to remove the calcium carbonate from the sample [280], which is also favorable.

More recently, Bradić et al. [281] developed a complete chitin extraction system using DESs based on [Ch]Cl as the HBA and lactic acid, malonic acid, urea or citric acid as the HBDs. [Ch]Cl:LA (1:1) allowed the best chitin extraction yield (85%) and chitin purity grade (98%), removing minerals and proteins in a one-step process. In this study, the recycling and re-use of the solvent was also evaluated. Two recycles of the solvent were performed and almost no loss in the chitin isolation yield was observed (only 2% loss). However, the solution became more viscous as contaminants such as minerals were accumulated [281].

Finally, DESs have been used for chitosan processing. Galvis-Sánchez et al. [282] prepared thermocompression molded films with chitosan (DA 90%), using [Ch]Cl:CA (1:1). SEM and XRD analysis revealed that acetic acid interacted with CA and promoted the reorganization of chitosan molecules and their amorphization, which in turn resulted in an increase in the elongation at break of the films. Moreover, in comparison to chitosan/CA films, chitosan/[Ch]:CA exhibited a stronger water sorption ability [282]. In a similar work, [Ch]Cl:malonic acid was used as a plasticizer for the production of chitosan-based films, leading to higher elongation at break [283]. 

[Ch]Cl-based DESs with urea and glycerol with and without co-solvents (DMF and water) have been applied as solvent media for chitosan methylation with methyl iodide. The use of DESs avoided polymer chain scission. It was verified that the different types of DES affected the type of methylation: [Ch]Cl:glycerol promoted O-methylation, while [Ch]Cl:U promoted N-methylation [282]. 

This research field is still very recent, and thus more developments are expected to arise in the near future to understand the dissolution phenomena and chitin extraction from biomass. The bulk of works regarding chitin dissolution is already significant for ILs; however, their application in selective extraction remains hindered due to their lack of selectivity and perhaps a similar approach to what has been done with hemicelluloses could be beneficial to solve this problem. Nonetheless, the industrial application of the IL [C_2_C_1_im][CH_3_COO] is a promising indication of the potential of these solvents not only for chitin but also for other polysaccharides’ extraction and dissolution. Regarding the use of DESs, while the solubility assays do not present promising results thus far, some interesting methodologies for chitin extraction from biomass have been presented with good results. This behavior could be due to the use of DESs to remove contaminants instead of selectively extracting chitin. Additionally, the synergy with other technologies seems essential in both cases to increase the extraction efficiency (MAE and UAE) or to help in the separation and purification of chitin (compressed CO_2_). Finally, in what partakes to chitosan, there are still very few works using DES and the focus is on their use as plasticizer agents.

## 7. Conclusions and Final Remarks

A review regarding the potentialities of green solvents, namely ILs and DESs, for polysaccharides dissolution and extraction from biomass, i.e., lignocellulosic biomass and food waste, was herein presented. There are indeed advantages in using ILs and DESs instead of volatile and non-environmentally friendly organic solvents or harsh acidic/alkaline solutions usually applied for polysaccharides dissolution and extraction.

There are relevant differences between the dissolution mechanisms of polysaccharides in ILs and DESs, which mainly depend on the physicochemical nature of the targeted polysaccharide, being the number of hydroxyl groups present in the polysaccharide structure, branching, degree of polymerization and composition. For instance, ILs demonstrated higher ability for cellulose dissolution in respect to DESs. One of the major conclusions reported in the literature lies in the highly ordered structure of ILs that is lost with cellulose. This allows increasing the entropy of the system and consequently enhances cellulose dissolution. The opposite is verified for DESs, which present a less ordered structure. In respect to starch dissolution, the use of ILs seems to bring new opportunities to tailor the physicochemical properties of this polysaccharide, where the IL concentration plays a crucial role, while the use of DESs seems to be more tailored to starch plasticization. On the other hand, there are still few studies addressing the dissolution and extraction of hemicelluloses and pectins with both types of solvents, but their application for this purpose seems promising, since the native structure of the polysaccharides seems almost preserved. The extraction of chitin from crustacean shells has been achieved successfully with both ILs and DESs and some of the designed processes allowed selective separation of chitin from the undesired proteins and inorganic fraction.

Although several studies are arising to understand the mechanisms behind and to accomplish the isolation of polysaccharides, there is still a counterbalance between efficiency and selectivity in the described extraction processes. Although ILs work very efficiently in polysaccharides dissolution, a major focus has been given to the entire biomass dissolution with the further selective precipitation of biomass components, including polysaccharides, through the addition of anti-solvents, instead of selective extraction. For some polysaccharides, namely hemicelluloses and starch, the application of ILs and DESs is more efficient and selective using water as a co-solvent. Moreover, there is a lack of studies focused on understanding the solubilization mechanisms first with model compounds, followed by the application of these solvents in extraction, which could be beneficial to attain promising results.

There is still the issue of the high price of ILs and DESs, and additional steps are required in the purification of fractionated samples. However, if properly designed and produced at a large scale, this problem can be overcome, as attested by the application of ILs at the industrial scale by the 525 Solutions company for the extraction of chitin using [C_2_C_1_im][CH_3_COO]. The field involving ILs is more complete since they have been studied in polysaccharides dissolution and extraction since 1934 [284], whereas the first work reporting DESs for the same purpose only appeared in 2001 [285]. Promising research has been done with DESs for polysaccharides solubilization and extraction, but this is a recent line of research that needs better understanding on the mechanisms underlying those processes. Although scarcely investigated, converging the advantages of these solvents with other technologies (MAE, UAE and UMAE) deserves special attention as it leads to higher extraction yields, while significantly reducing time. On the other hand, the recycling of ILs and DESs has been seldom attempted; yet, approaches such as the use of supercritical solvents and precipitation of the extracted polysaccharides using anti-solvents such as water and ethanol have been proposed.

Albeit more focused research is needed to better understand the dissolution mechanisms and thus design solvents with high selectivity and extraction efficiency, both ILs and DESs share high potential to be implemented and integrated in biorefinery platforms towards a more sustainable biomass valorization and to meet the desired bioeconomy concept.

## Figures and Tables

**Table 1:** Chemical and structural characteristics of polysaccharides mentioned in this work.

Polysaccharide	Source	Monomer	Linkages	DP	Structure	Ref.
Cellulose	Lignocellulosic biomass	d-glucose	β(1→4)	500–10,000	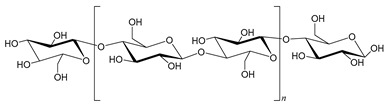	[24,27]
Hemicelluloses	Lignocellulosic biomass	β-d-xylose, α-l-arabinose, β-d-glucose, α-d-galactose, β-d-mannose. α-d-glucuronic, α-d-galacturonic, α-d-4-*O*-methylgalacturonic acids	β(1→4)	80–200	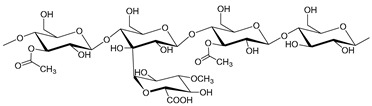	[3,38,40]
Starch	Endosperm and plant tubers	d-glucose	α(1→4) α(1→6) (amylopectin)	6000 (amylose) 2 million (amylopectin)	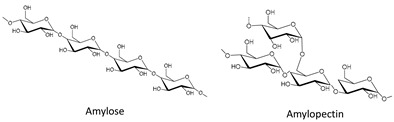	[55,80,81]
Pectins	Fruit peels and pulp	α-d-galacturonic acid α-l-arabinose, β-d-galactose, α-l-α-rhamnose and β-d-xylose (ramifications)	α(1→4)	Up to 1000	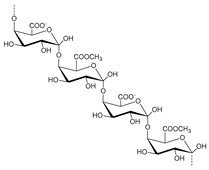	[63,64,82]
Chitin	Crustaceans shells, insect exoskeleton and fungi cell walls	2-acetamido-2-deoxy-β-d-glucose	β(1→4)	2000–4000	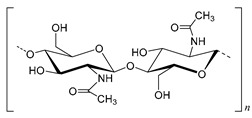	[23,69,70]

**Table 2:** Name and respective acronym of cation–anion combinations in ionic liquids (ILs).

Cation			Anion		
	Name	Acronym		Name	Acronym
i.	1-alkylimidazolium	[C*_n_*im]^+^	i.	chloride	Cl^−^
ii.	1-alkyl-3-methylimidazolium	[C*_n_*C_1_im]^+^	ii.	bromide	Br^−^
iii.	1-allyl-2,3-dimethylimidazolium	[aC_1_C_1_im]^+^	iii.	fluoride	F^−^
iv.	1,2-alkyl-3-methylimidazolium	[C*_n_*C*_n_*C_1_im]^+^	iv.	iodide	I^−^
v.	1-(3,6-dioxalkyl)-3-alkylimidazolium	[C_1_(OC_2_)*_n_*C*_n_*im]^+^	v.	thiocyanate	[SCN]^−^
vi.	1-(2-hydroxyethyl)-3-methylimidazolium	[C_2_OHC_1_im]^+^	vi.	tetrafluoroborate	[BF_4_]^−^
vii	1-(4,8,12-trioxatridecyl)-3-ethylimidazolium	[C_1_(OC_3_)_3_C_2_im]^+^	vii	hexafluorophosphate	[PF_6_]^−^
viii	1-(3,6-dioxaheptyl)-3-(3,6,9-trioxadecyl)imidazolium	[C_1_(OC_2_)_3_C_1_OC_2_OC_1_im]^+^	viii	bis(trifluoromethylsulfonyl)imide	[NTf_2_]^−^
ix.	1-(1-cyanoethyl)-3-methylimidazolium	[CNC_2_C_1_im]^+^	ix.	dicyanamide	[N(CN)_2_]^−^
x.	1-benzyl-2,3-alkylimidazolium	[BzC*_n_*C*_n_*im]^+^	x.	hydroxide	[OH]^−^
xi.	tetrabutylammonium	[N4444]^+^	xi.	formate	[HCOO]^−^
xii.	*N*-allyltrialkylammonium	[Na*nnn*]^+^	xii.	acetate	[CH_3_COO]^−^
xiii.	dimethylalkylammonium	[N11*n*]^+^	xiii.	propanoate	[CH_3_CH_2_COO]^−^
xiv.	benzyldimethyl(alkyl)ammonium	[N11*n*Bz]^+^	xiv.	butanoate	[CH_3_CH_2_CH_2_COO]^−^
xv.	triallylbenzylammonium	[N(a)_3_Bz]^+^	xv.	benzoate	[(C_6_H_5_)COO]^−^
xvi.	*N*-*N*-dimethylethanolammonium	[N11(C_2_OH)]^+^	xvi.	glycolate	[HOCH_2_COO]^−^
xvii.	*N*-methyl-*N,N*-bis(2-methoxyethyl)ammonium	[N1(C_1_OC_2_)_2_]^+^	xvii.	thioglycolate	[HSCH_2_COO]^−^
xviii.	*N,N*-bis(2-methoxyethyl)ammonium	[N(C_1_OC_2_)_2_]^+^	xviii.	lactate	[CH_3_CHOHCOO]^−^
xix.	*N,N*-dimethyl-2-methoxyethylammonium	[N11(C_1_OC_2_)]^+^	xix.	aminoethanoate	[H_2_NCH_2_COO]^−^
xx.	*N*-*n*(2-methoxyethyl)-*N*-methyl-*N*-propylammonium	[N13(C_1_OC_2_)*_n_*]^+^	xx.	phosphate	[PO_4_]^−^
xxi.	AMMOENG110	[Amm110]^+^	xxi.	dialkylphosphate	[(C*_n_*O)_2_PO_2_]^−^
xxii.	*N*-allyl-*N*-methylmorpholinium	[aC_1_mor]^+^	xxii.	methylphosphinate	[(C_1_O)HPO_2_]^−^
xxiii.	*N*-alkyl-*N*-methylmorpholinium	[C*_n_*C_1_mor]^+^	xxiii.	alkylphosphonate	[(C*_n_*O)C*_n_*PO_2_]^−^
xxiv.	*N*-allyl-*N*-methylpiperidium	[aC_1_pip]^+^	xxiv.	hydrogen sulfate	[HSO_4_]^−^
xxv.	*N*-butyl-*N*-methylpiperidium	[C_4_C_1_pip]^+^	xxv.	triflate	[TfO]^−^
xxvi.	methylalkylalkylpiperidium	[C_1_C*_n_*C*_n_*pip]^+^	xxvi.	methanosulfonate	[MsO]^−^
xxvii.	tetralkylphosphonium	[P*_nnnn_*]^+^	xxvii.	alanilate	[Ala]
xxviii.	3-alkyl-*N*-alkylpyridinium	[C*_n_*C*_n_*py]^+^	xxviii.	serinate	[Ser]
xxix.	alkyl-alkylpyrrolidinium	[C*_n_*C*_n_*pyr]^+^	xxix.	cysteinate	[Cys]^−^
xxx.	1,1,3,3-tetramethylguanidine	[TMGH]^+^	xxx.	prolinate	[Pro]^−^
xxxi.	diethylmethylsulphonium	[EMS]^+^	xxxi	aspartinate	[Asp]^−^
xxxii.	cholinium	[Ch]^+^		valinate	[Val]^−^
				leucinate	[Leu]^−^
				phenilalanilate	[Phe]^−^

**Table 3:** Name and respective acronym of the hydrogen bond acceptor (HBA)–hydrogen bond donor (HBD) combinations in deep eutectic solvents (DESs).

HBA			HBD		
	Name	Acronym		Name	Acronym
i.	cholinium chloride	[Ch]Cl	i.	lactic acid	LA
ii.	chlorocholinium chloride	[ClCh]Cl	ii.	malonic acid	--
iii.	cholinium bromide	[Ch]Br	iii.	malic acid	MA
iv.	cholinium lactate	[Ch][CH_3_CHOHCOO]	iv.	citric acid	CA
v.	cholinium acetate	[Ch][CH_3_COO]	v.	Oxalic acid	--
vi.	allyltrimethylammonium chloride	[Na111]Cl	vi.	urea	U
viii.	tetrabutylammonium hydroxide	[N4444]OH	vii.	*N*-methylurea	--
viii.	1-methyl-2-oxopyrrolidinium chloride	[C1Opyr]Cl	viii.	thiourea	--
ix.	nicotinic acid	--	ix.	1,1-dimethylurea	--
x.	urea	U	x.	glycerol	--
xi.	betaine	--	xi.	imidazole	im
xii.	glycine	--	xii.	zinc chloride	ZnCl_2_
xiii.	alanine	--	xiii.	potassium hydroxide	KOH
xiv.	histidine	--	xiv.	glucose	--
xv.	glucose	--			

**Table 4:** ILs reported to dissolve cellulose, cellulose solubility and conditions applied.

Cation	Anion	Cellulose Type	Conditions	Solubility (wt%)	References
[C*_n_*C_1_im]^+^	Cl^−^	Avicel α-Cellulose Cotton linters Cellulose dissolving pulp Kraft pulp Commercial cellulose Eucalyptus pre-hydrolysis sulfate pulp Spruce sulfite pulp MCC	*T* = 70–130 °C With and without sonication	0.5–14 >5 4–10 0.1–10 9 1.8 15.8 6 8	[132,133,155,156,157,158,159,160,161]
	Br^−^	Avicell Pulp cellulose ^a^ Commercial cellulose ^b^	*T* = 80–100 °C Microwave heating	1–3 5–7 1.7–3.4	[133,157,160]
	[BF_4_]^−^	Cellulose dissolving pulp	Microwave heating	Insoluble	[133]
	[PF_6_]^−^	Cellulose dissolving pulp	Microwave heating	Insoluble	[133]
	[CH_3_COO]^−^	Cellulose ^a^ Avicell α-Cellulose Eucalyptus pre-hydrolysis sulfate pulp MCC	*T* = 70–120 °C	>20 <1–15 >5 19.6–18.6 29.1	[141,157,158,162,163,164]
	[(CO)*_n_*C*_n_*PO_2_]^−^	Avicel α-Cellulose MCC	*T* = 100–55 °C	5–10	[165]
	[(C_1_O)HPO_2_]^−^	MCC	*T* = 25–45 °C	4–10	[165]
	[(C_2_O)_2_PO_2_]^−^	Avicel	*T* = 100 °C	12–14	[165]
	[HSCH_2_COO]^−^	MCC	*T* = 70 °C	13.5	[165]
	[HCOO]^−^	Avicel MCC	*T* = 110 °C *T* = 70 °C	8 12.5	[163,164]
	[(C_6_H_5_)COO]^−^	MCC	*T* = 70 °C	12	[164]
	[H_2_NCH_2_COO]^−^	MCC	*T* = 70 °C	12	[164]
	[HOCH_2_COO]^−^	MCC	*T* = 70 °C	10.5	[164]
	[CH_3_CHOHCOO]^−^	MCC	*T* = 70 °C	9.5	[164]
	I^−^	Avicel	*T* = 100 °C	1–2	[157]
	F^−^	Avicel	*T* = 100 °C	2	[157]
	[N(CN)_2_]^−^	Avicel MCC	*T* = 110 °C *T* = 70 °C	1 0	[163,164]
	[NTf_2_]^−^	Avicel	*T* = 110 °C	<0.5	[164]
[aC_1_C_1_im]^+^	Br^−^	Avicel Cotton linters Spruce sulfite pulp	--	12 4 4	[156]
[aC_1_im]^+^	Cl^−^	Avicel α-Cellulose Cotton linters Pulp cellulose ^a^ Kraft pulp Eucalyptus pre-hydrolysis sulfate pulp Spruce sulfite pulp MCC	*T* = 80–100 °C With and without sonication	20–18 >5 13 14.5–25 8 13.6 13 27	[159,166,167,168]
	[HCOO]^−^	MCC	*T* = 85 °C	22	[168]
[C*_n_*C*_n_*C_1_im]^+^	Cl^−^	Avicel Cotton linters Eucalyptus pre-hydrolysis sulfate pulp Spruce sulfite pulp	*T* = 80–120 °C	9 4 12.8 6	[132,156,158]
	[CH_3_COO]^−^	MCC	*T* = 40–120 °C	<0.3–17.3	[141]
[C_2_OHC_1_im]^+^	[CH_3_COO]^−^	MCC	*T* = 40–120 °C	0.5–18.1	[141]
[C_1_OC_2_C_1_im]^+^	[CH_3_COO]^−^	MCC	*T* = 40–120 °C	3.9–27.6	[141]
[C_1_(OC_2_)_2_C_2_im]^+^	Cl^−^	Avicel	*T* = 110 °C	2	[163,169]
	[CH_3_COO]^−^	Avicel	*T* = 110 °C	12	[163,169]
[C_1_(OC_2_)_3_C_2_im]^+^	[CH_3_COO]^−^	Avicel	*T* = 110 °C	12	[163,169]
[C_1_(OC_2_)_4_C_2_im]^+^	[CH_3_COO]^−^	Avicel	*T* = 110 °C	10	[163,169]
[C_1_(OC_2_)_7_C_2_im]^+^	[CH_3_COO]^−^	Avicel	*T* = 110 °C	3	[163,169]
[H(OC_2_)_2_C_1_im]^+^	Cl^−^	Avicel	*T* = 110 °C	1	[163]
	[CH_3_COO]^−^	Avicel	*T* = 110 °C	5	[163]
[H(OC_2_)_3_C_1_im]^+^	[CH_3_COO]^−^	Avicel	*T* = 110 °C	2	[163,169]
[C_1_(OC_3_)_3_C_2_im] ^+^	[CH_3_COO]^−^	Avicel	*T* = 110 °C	0.5	[163,169]
[C_1_(OC_2_)_3_C_4_im]^+^	[CH_3_COO]^−^	Avicel	*T* = 110 °C	0.5	[163,169]
[C_1_(OC_2_)_3_C_1_O C_2_OC_1_im]^+^	[CH_3_COO]^−^	Avicel	*T* = 110 °C	0.5	[163,169]
[CNC_1_im]^+^	Br^−^	Commercial cellulose ^b^	*T* = 80–90 °C	3.4	[160]
[BzC_1_im]^+^	[CH_3_COO]^−^	MCC	*T* = 40–120 °C	0.4–34	[142]
[BzC_4_C_2_im]^+^	[CH_3_COO]^−^	MCC	*T* = 40–120 °C	<0.3	[142]
[N13(C_1_OC_2_)*_n_*]^+^	[CH_3_COO]^−^	Avicel	*T* = 110 °C	3–10	[163]
[N11(C_2_OH)]^+^	[CH_3_COO]^−^	Avicel	*T* = 110 °C	<0.5	[163]
[N1(C_1_OC_2_)_2_]^+^	[CH_3_COO]^−^	Avicel	*T* = 110 °C	<0.5	[163]
[N(C_1_OC_2_)_2_]^+^	[CH_3_COO]^−^	Avicel	*T* = 110 °C	<0.5	[163]
[N11(C_1_OC_2_)]^+^	[CH_3_COO]^−^	Avicel	*T* = 110 °C	<0.5	[163]
[N_4444_]^+^	[HCOO]^−^	Avicel	*T* = 110 °C	1.5	[163]
[N_1114Bz_]^+^	Cl^−^	Avicel Cotton linters Pulp cellulose ^a^	*T* = 62 °C	5 1 2	[132,154]
[N_a444_]^+^	[CH_3_COO]^−^	Cellulose ^a^	*T* = 100 °C	2	[170]
[N_a222_]^+^	[CH_3_COO]^−^	Cellulose ^a^	*T* = 100 °C	2	[170]
[N_a111_]^+^	[CH_3_COO]^−^	Cellulose ^a^	*T* = 100 °C	2	[170]
[Amm110]^+^	[HCOO]^−^	Avicel	*T* = 110 °C	0.5	[164]
	Cl^−^	Avicel	*T* = 110 °C	0.5	[164]
	[N(CN)_2_]^−^	Avicel	*T* = 110 °C	> 0.5	[164]
	[CH_3_COO]^−^	Avicel	*T* = 110 °C	0.5	[164]
[N_11*n*_]	[CH_3_COO]^−^	Avicel	*T* = 100 °C	9	[171]
[aC_1_mor]^+^	[CH_3_COO]^−^	MCC Cell-A (DP = 1644) Cell-M (DP = 2082) MCC	*T* = 80–120 °C *T* = 40–120 °C	17–30 13–28 11-250.5–14.7	[155,171] [141]
	[PO_4_]^−^	Cellulose ^a^	*T* = 80 °C	2	[170]
	[HCOO]^−^	Cellulose ^a^	*T* = 80 °C	2	[170]
[C_4_C_1_mor]^+^	[CH_3_COO]^−^	Cellulose ^a^	*T* = 80 °C	2	[170]
[aC_1_pip]^+^	[CH_3_COO]^−^	MCC	*T* = 40–120 °C	5–10.0	[141]
[C_4_C_1_pip]^+^	Cl^−^	Cotton linters α-Cellulose	*T* = 105 °C *T* = 105 °C	12 5	[168]
	[CH_3_COO]^−^	MCC	*T* = 40–120 °C	0.6–4.4	[141]
[P_4444_]^+^	[HCOO]^−^	Avicel	*T* = 110 °C	6	[163]
[P_66614_]^+^	[N(CN)_2_]^−^	Avicel	*T* = 110 °C	<2.8	[164]
[C_4_C_1_py]^+^	Cl^−^	Avicel α-Cellulose Cotton linters Spruce sulfite pulp	*T* = 105 °C	39 >5 12 37	[132,154,168]
[TMG]^+^	[HCOO]^−^	MCC	*T* = 100 °C	5	[148]
	[CH_3_COO]^−^	MCC Eucalyptus PHK-dissolving pulp	*T* = 100 °C *T* = 80 °C	5 5	[148] [172]
	[CH_3_CH_2_COO]^−^	MCC Eucalyptus PHK-dissolving pulp	*T* = 100 °C *T* = 80 °C	5 15	[148] [172]
	[CH_3_CH_2_CH_2_COO]^−^	MCC	*T* = 100 °C	5	[148]

^a^ Not specified. ^b^ Fibers between 0.02 and 1.5 mm diameter.

**Table 5:** Mixtures of ILs/co-solvents reported to dissolve cellulose, cellulose solubility and conditions applied.

Co-Solvent	Cation	Anion	Cellulose Type	Conditions	Solubility (wt%)	References
DMSO	[C_4_C_1_im]^+^	[CH_3_COO]^−^	Avicel	60 °C	16.0	[177]
	[C_3_OC_1_im]^+^	[CH_3_COO]^−^			13.0	
	[C_1_C_1_im]^+^	[(C_1_O)_2_PO_2_]^−^	MCC	100 °C	5.0	[178]
	[C_2_C_1_im]^+^					
	[C_4_C_1_im]^+^					
	[C_6_C_1_im]^+^					
	[NBz111]^+^	[CH_3_COO]^−^	MCC	60 °C	<0.5	[174]
	[NaBz_1_]^+^	[CH_3_COO]^−^			6	
	[NBz_3_1]^+^	[CH_3_COO]^−^			4	
	[N4444]^+^	[CH_3_COO]^−^			8	
	[NaBz11]^+^	[CH_3_COO]^−^	MCC	60 °C	10	[175]
	[Na_2_Bz1]^+^	[CH_3_COO]^−^			10	
	[Na_3_Bz]^+^	[CH_3_COO]^−^			10	
	[Na_2_1]^+^	[CH_3_COO]^−^			3	
	[NBz_3_1]^+^	[CH_3_COO]^−^			3	
	[N4444]^+^	[CH_3_COO]^−^			15	
	[N4444]^+^	[CH_3_COO]^−^	MCC	60 °C	12	[185]
1-alkylimidazole	[C_2_C_1_im]^+^	[(C_1_O)HPO_2_]^−^	Cotton fibers	MW pulses 90 °C	5.0	[176]
	[C_4_C_1_im]^+^				<5.0	
GVL	[C_2_C_1_im]^+^	Cl^−^	α-Cellulose	80 °C	15.0	[175]
	[C_4_C_1_im]^+^	Cl^−^			13.0	
	[aC_1_im]^+^	CH_3_COO^−^			7.0	
DMF	[C_3_(SO_3_H)C_2_OC_1_im]^+^	[HSO_4_]^−^ [H_2_PO_4_]^−^ [TfO]^−^ [MsO]^−^	MCC	100 °C	15.1 16.1 16.4 15.4	[173]
	[C_3_(SO_3_H)C_2_OC_2_im]^+^	[HSO_4_]^−^ [H_2_PO_4_]^−^ [TfO]^−^ [MsO]^−^			19.8 19.6 18.8 18.5	
	[C_3_(SO_3_H)(C_2_O)_2_C_1_im]^+^	[HSO_4_]^−^ [H_2_PO_4_]^−^ [TfO]^−^ [MsO]^−^			10.9 9.7 14.5 13.9	
	[C_3_(SO_3_H)(C_2_O)_2_C_1_im]^+^	[HSO_4_]^−^ [H_2_PO_4_]^−^ [TfO]^−^ [MsO]^−^			9. 8.2 12.7 13.4	
	[C_1_C_1_im]^+^	[(C_1_O)_2_PO_2_]^−^	MCC	100 °C	5.0	[178]
	[C_2_C_1_im]^+^					
	[C_4_C_1_im]^+^					
	[C_6_C_1_im]^+^					

**Table 6:** ILs reported to dissolve starch, starch solubility and conditions applied.

Cation	Anion	Starch Type	Conditions	Solubility (wt%)	References
[im]^+^	[HCOO]^−^	Barley starch	*T* = 80 °C (catalyst *p*-TsOH ^a^)	10	[204]
[C_1_im]^+^	[HCOO]^−^	Barley starch	*T* = 80 °C (catalyst *p*-TsOH ^a^)	10	[204]
[C_4_im]^+^	[HCOO]^−^	Barley starch	*T* = 80 °C (catalyst *p*-TsOH ^a^)	10	[204]
[C_1_C_1_im]^+^	[(C_1_O)HPO_2_]^−^	Maize starch	*T* = 80 °C	10	[214]
[C_2_C_1_im]^+^	[CH_3_COO]^−^	Maize starch (24.4 wt% amylose) Corn starch Waxy corn starch	Room Temperature *T* ≥ 36 °C *T* = 80 °C	10 20 10	[203] [51] [204,215]
	[(C_1_)_2_PO_4_]^−^	Barley starch	*T* = 80 °C (catalyst *p*-TsOH ^a^	10	[204]
[C_4_C_1_im]^+^	Cl^−^	High amylose maize starch Waxy maize starch Barley starch	*T* = 105 °C *T* = 80 °C *T* = 100 °C *T* = 80 °C (catalyst *p*-TsOH ^a^)	9.5 15 2 10	[206] [52,207] [216] [204]
	Br^−^	Barley starch	*T* = 80 °C (catalyst *p*-TsOH ^a^)	10	[204]
	[N(CN)_2_]^−^	--	*T* = 90 °C	10	[52]
[C_6_C_1_im]^+^	Cl^−^	Barley starch	*T* = 80 °C (catalyst *p*-TsOH ^a^)	10	[204]
	Br^−^	Barley starch	*T* = 80 °C (catalyst *p*-TsOH ^a^)	10	[204]
[aC_1_im]^+^	Cl^−^	Corn starch	*T* = 80 and 100 °C Argon atmosphere	20	[204,207]
[Ch]^+^	[CH_3_COO]^−^	Corn starch	*T* ≥ 68 °C	20	[51]
[N(C_2_OH)]^+^	[HCOO]^−^	Barley starch	*T* = 80 °C (catalyst *p*-TsOH ^a^)	10	[204]

^a^*p-*TsOH-*p*-Toluenesulfonic acid.

**Table 7:** DESs reported to dissolve starch, starch solubility and conditions applied.

HBA	HBD	Molar Ratio	Starch Type	Conditions	Solubility (wt%)	References
[Ch]Cl	Urea	1:1 ^a^	n.d.	*T* = 100 °C	9.1	[52]
	Glucose	1:1	n.d.	--	17.2 ^b^	[218]
	ZnCl_2_	1:1.9	n.d.	*T* = 98 °C	4.6	[52]
	Imidazole	3:7	Potato	*T* = 100 °C	20.0	[113]
	Malic Acid	1:1	n.d.	*T* = 100 °C	7.10	[217]
	Lactic Acid	1:10	n.d.	*T* = 60 °C	0.13	[217]
	Citric Acid	1:1.4 ^a^	n.d.	*T* = 100 °C	8.3	[52]
	Urea:Glycerol	1:1:1	Potato	*T* = 130 °C	10	[221]
	Oxalic Acid	1:1	n.d.	*T* = 60 °C	0.15	[217]
		1:1.6 ^a^	n.d.	*T* = 60 °C	9.1	[52]
[Ch][C_2_OCO_2_]	Urea	1:2	Potato	*T* > 110 °C	10	[221]
[Ch][CH_3_COO]	Urea	1:2	Potato	*T* > 110 °C	10	[221]
[C_1_Opyr]Cl	Oxalic Acid	1:1	n.d.	Room Temperature	10	[217]
Nicotinic Acid		1:9	n.d.	*T* = 60 °C	2.83	[217]
Urea	CaCl_2_	1:4 ^a^	n.d.	*T* = 80 °C	16.7	[52]
Betaine	Malic Acid	1:1	n.d.	*T* = 100 °C	0.81	[217]
Glycine		1:1	n.d.	*T* = 100 °C	7.65	[217]
Proline		3:1	n.d.	*T* = 100 °C	5.90	[217]
		2:1	n.d.	*T* = 100 °C	0.32	[217]
Alanine		1:1	n.d.	*T* = 100 °C	0.29	[217]
	Lactic Acid	1:9	n.d.	*T* = 60 °C	0.26	[217]
Glucose		1:5	n.d.	*T* = 100 °C	1.67	[220]
Histidine		1:9	n.d.	*T* = 60 °C	0.13	[217]

^a^ Weight ratio instead of molar ratio. ^b^ mg/mL instead of wt%. n.d.—not disclosed.

**Table 8:** ILs reported to extract pectin, pectin yield and experimental conditions applied.

Cation	Anion	Biomass Type	Conditions	Yield (wt%)	References
[Ch]^+^	[Leu]^−^	Ponkan peels	S/L = 1 g/17 g 100 min *T* = RT ^a^	13.42	[246]
[SO_3_HC_4_C_1_im]^+^	[HSO_4_]^−^	Pomelo peels	S/L = 2 g/ 27 mL 15 min 360 W (UMAE)	328.64 ^b^	[244]
[C_2_C_1_im]^+^	Br^−^	Lemon peels	S/L = 1 g/15 mL 5 min *T* = 80 °C MAE	10.8	[247]
	[BF_4_]^−^			7.8	
[C_4_C_1_im]^+^	Cl^−^		S/L = 1 g/22.7 mL 9.6 min *T* = 88 °C MAE	24.7	
	Br^−^		S/L = 1 g/15 mL 5 min *T* = 80 °C MAE	15.7	
	[BF_4_]^−^			11.2	
[aC_1_im]	Cl^−^			12.1	

^a^ Room temperature. ^b^ mg/g.

**Table 9:** DESs reported to extract pectin, pectin yield and experimental conditions applied.

HBA	HBD	Biomass Type	Conditions	Yield (wt%)	References
Glucose	Lactic Acid	Pomelo peels	S/L = 1 g/29 mL 141 min T = 88 °C	7.39–23.04 ^a^	[248]
Glycine	Lactic Acid		S/L= 1 g/29 mL 141 min T = 88 °C	Gelation	
[Ch]Cl	Malonic Acid	Pomelo peels	S/L = 1 g/40 mL 120 min (UAE) T = 60 °C	93.37	[249]
	Citric Acid			43.18	
	Malic Acid			44.96	
	Oxalic Acid			32.91	
	Frutose			29.25	
	Glycerol			41.71	
	Glucose			96.73	
	Saccharose			51.73	
Frutose	Citric Acid			36.97	

^a^ Varying DESs wt.% in water.

**Table 10:** ILs reported to dissolve chitin and chitosan, their solubility and conditions applied.

Cation	Anion	Polysaccharide	Conditions	Solubility (wt%)	References
[aC_1_im]^+^	Br^−^	Chitin	T = 100 °C	2 5 10 >5.0	[267] [268] [269] [270]
	Cl^−^		T = 110 °C T < 45 °C	Insoluble 0.5	[259] [271]
[C_2_C_1_im]^+^	[HCOO]^−^		T = 110 °C	Insoluble	[259]
	[CH_3_COO]^−^		T = 100 °C (or MW) T = 105 °C T = 100 °C	20 9 >5	[258] [272] [270]
	Cl^−^		T = 100 °C (or MW) T = 105 °C	3.5 3.0	[258] [273]
	Br^−^		T > 87 °C T = 105 °C	Insoluble 12.0	[274] [273]
	I^−^		T = 105 °C	11.0	[273]
	[BF_4_]^−^		T > 87 °C	Insoluble	[274]
	[TfO]^−^		T > 87 °C	Insoluble	[274]
	[CH_3_CHOHCOO]^−^		T = 105 °C	8	[272]
[HOC_2_C_1_im]^+^	Cl^−^		T = 100 °C	>5	[270]
[C_4_C_1_im]^+^	[CH_3_COO]^−^		T = 100 °C	6 3 ^a^	[259] [265]
	Cl^−^		T = 110 °C T = 110 °C T = 100 °C (or MW) T = 100 °C	<1 10 6 Partially soluble	[259] [275] [258] [276]
	Br^−^		T = 100 °C	<1	[268]
[C_1_C_3_pyr]^+^	[CH_3_COO]^−^		T = 105 °C	1.2	[272]
	[CH_3_CHOHCOO]^−^		T = 105 °C	4.2	[272]
[C_2_pyr]^+^	I^−^		T = 105 °C	6.0	[273]
[C_1_C_3_pip]^+^	[CH_3_COO]^−^		T = 105 °C	2.8	[272]
	[CH_3_CHOHCOO]^−^		T = 105 °C	1.6	[272]
[C_1_C_3_C_1_pip]^+^	[CH_3_COO]^−^		T = 105 °C	5.0	[272]
	[CH_3_CHOHCOO]^−^		T = 105 °C	2.6	[272]
[EMS]^+^	[NTf_2_]^−^		T = 105 °C	<1.0	[273]
[N_4444_]^+^	[OH]^−^		T = 90 °C	1.59	[277]
[C_2_C_1_im]^+^	[CH_3_COO]^−^	Chitosan	T = 40–140 °C	0.6–13.8	[256]
[C_4_C_1_im]^+^	[HCOO]^−^		T = 110 °C T = 50–150 °C	12 0.6–8.4	[259] [262]
	[CH_3_COO]^−^		T = 110 °C T = 50–150 °C T = 40–140 °C	12 0.8–14.4 0.6–13.8	[259] [262] [256]
	[CH_3_CH_2_COO]^−^		T = 50–150 °C	0.2–12.4	[262]
	[CH_3_CH_2_CH_2_COO]^−^		T = 50–150 °C	0.2–10.4	[262]
	[HOCH_2_COO]^−^		T = 50–150 °C	0.6–9.6	[262]
	[CH_3_CHOHCOO]^−^		T = 50–150 °C	0.4–6.2	[262]
	[C_6_H_5_COO]^−^		T = 50–150 °C	1.4–7.6	[262]
	[N(CN)_2_]^−^		T = 50–150 °C	Insoluble	[262]
	Cl^−^		T = 110 °C	10	[275]
[C_6_C_1_im]^+^	[CH_3_COO]^−^		T = 40–140 °C	0.6–12	[256]
[C_8_C_1_im]^+^	[CH_3_COO]^−^		T = 40–140 °C	0.2–7.4	[256]
[C_4_C_1_C_1_im]^+^	[CH_3_COO]^−^		T = 40–140 °C	6.4−>9.8	[256]
[N_2222_]^+^	[CH_3_COO]^−^		T = 40–140 °C	0.4–0.8	[256]
[N_22_]^+^	[CH_3_COO]^−^		T = 40–140 °C	Insoluble	[256]
[N(C_3_OC_1_)_2_]^+^	[CH_3_COO]^−^		T = 40–140 °C	0.5−>3.6	[256]
[pyr]^+^	[CH_3_COO]^−^		T = 40–140 °C	0.1–1.3	[256]

^a^ (*w/v*)%.
